# Anthelmintic Activity and Cytotoxic Effects of Compounds Isolated from the Fruits of *Ozoroa insignis* Del. (Anacardiaceae)

**DOI:** 10.3390/biom11121893

**Published:** 2021-12-17

**Authors:** Mthandazo Dube, Mohamad Saoud, Robert Rennert, Ghislain Wabo Fotso, Kerstin Andrae-Marobela, Peter Imming, Cécile Häberli, Jennifer Keiser, Norbert Arnold

**Affiliations:** 1 Department of Bioorganic Chemistry, Leibniz Institute of Plant Biochemistry, D-06120 Halle (Saale), Germany; Mthandazo.Dube@ipb-halle.de (M.D.); Mohamad.Saoud@ipb-halle.de (M.S.); Robert.Rennert@ipb-halle.de (R.R.); 2 Department of Organic Chemistry, Faculty of Science, University of Yaoundé 1, Yaoundé P.O. Box 812, Cameroon; ghis152001@gmail.com; 3 Department of Biological Sciences, Faculty of Science, University of Botswana, Gaborone P.O. Box 0022, Botswana; k_marobela@yahoo.com; 4 Institute of Pharmacy, Faculty of Natural Sciences, Martin-Luther-University Halle-Wittenberg, D-06120 Halle (Saale), Germany; peter.imming@pharmazie.uni-halle.de; 5 Swiss Tropical and Public Health Institute, CH-4051 Basel, Switzerland; cecile.haeberli@swisstph.ch (C.H.); jennifer.keiser@swisstph.ch (J.K.); 6 University of Basel, CH-4051 Basel, Switzerland

**Keywords:** *Ozoroa insignis* Del., fruits, anthelmintic properties, cytotoxicity, *Caenorhabditis elegans*, parasitic worms

## Abstract

*Ozoroa insignis* Del. is an ethnobotanical plant widely used in traditional medicine for various ailments, including schistosomiasis, tapeworm, and hookworm infections. From the so far not investigated fruits of *Ozoroa insignis*, the anthelmintic principles could be isolated through bioassay-guided isolation using *Caenorhabditis elegans* and identified by NMR spectroscopic analysis and mass spectrometric studies. Isolated 6-[8(*Z*)-pentadecenyl] anacardic (**1**), 6-[10(*Z*)-heptadecenyl] anacardic acid (**2**), and 3-[7(*Z*)-pentadecenyl] phenol (**3**) were evaluated against the 5 parasitic organisms *Schistosoma mansoni* (adult and newly transformed schistosomula), *Strongyloides ratti*, *Heligmosomoides polygyrus*, *Necator americanus*, and *Ancylostoma ceylanicum*, which mainly infect humans and other mammals. Compounds **1**–**3** showed good activity against *Schistosoma mansoni*, with compound **1** showing the best activity against newly transformed schistosomula with 50% activity at 1µM. The isolated compounds were also evaluated for their cytotoxic properties against PC-3 (human prostate adenocarcinoma) and HT-29 (human colorectal adenocarcinoma) cell lines, whereby compounds **2** and **3** showed antiproliferative activity in both cancer cell lines, while compound **1** exhibited antiproliferative activity only on PC-3 cells. With an IC_50_ value of 43.2 µM, compound **3** was found to be the most active of the 3 investigated compounds.

## 1. Introduction

Human nematode infections are the most prevalent infections, affecting 1.5 billion people or 24% of the world’s population [1]. Despite this high global prevalence, medical research into parasitic diseases remains neglected. In Africa, multiple ethnic groups have used traditional medicine for centuries to treat nematode infections [2]. *Ozoroa insignis* Del. (Anacardiaceae) is used as a traditional medicinal plant for many indications, including intestinal worms. For example, in Botswana, people use a decoction of the roots or add fresh crushed fruits to porridge to eliminate the parasites (J. Mlilo, personal communication, 17 December 2019). In spite of this information, we did not find any literature pertaining to the anthelmintic properties or chemical constituents of the fruits of *O. insignis*.

The anthelmintic effect of the roots, leaves, and bark of *O. insignis*, but not the fruits, have been evidenced in vitro against *Schistosoma mansoni* and *Hymenolepis diminuta* [3]. Additionally, the bark has also shown in vitro cytotoxic activity against HEP-G2 (human hepatocellular carcinoma), MDA-MB-231 (human mammary adenocarcinoma), and 5637 (human bladder carcinoma) cell lines [4]. In order to isolate the active phytoconstituents of *O. insignis*, studies have been performed on different organs of the plant, which led to the identification of a variety of structurally diverse metabolites. From the roots of the plant, several tirucallane triterpenes have been recognized [5]. From the same organ, a macrolide named ozoroalide was isolated [6]. Several closely related anacardic acids (compounds consisting of a salicylic acid moiety with an additional saturated or unsaturated alkyl chain) were recognized from *O. insignis*. From the twigs, the anarcadic acid derivative, 6-pentadecylsalicylic acid, was isolated [7]. Furthermore, from the root bark of the plant 6-tridecyl anacardic acid, 6-[8(*Z*)-pentadecenyl] anacardic acid, 6-[10(Z)-heptadecenyl] anacardic acid, and 6-[nonydecyl] anacardic acid, a flavonoid named ozoranone, as well as triterpenes, for instance, β-amyrin, magnificol, betulonic acid, and betulinic acid were isolated [3,4]. Other chemical investigations have shown that plants containing anacardic acids like *O. insignis* commonly have a mixture of related phenolic lipids including cardols and cardanols [8]. To the best of our knowledge, there is no report on the constituents of *O. insignis* fruits. We herein report the identification of 6-[8*(Z)*-pentadecenyl] anacardic acid (**1**), trivially named ginkgolic acid C15:1 (CAS 22910-60-7), 6-[10(*Z*)-heptadecenyl] anacardic acid (**2**), trivially named ginkgolic acid C17:1 (CAS 111047-30-4) and 3-[7(*Z*)-pentadecenyl] phenol (**3**; CAS 936109-15-8) from the fruits of *O. insignis* through bioassay-guided isolation using the well-established test system with the model organism *Caenorhabditis elegans* [9,10,11]. The anthelmintic properties of **1**–**3** were next evaluated against the 5 parasitic organisms *Schistosoma mansoni* (adult and newly transformed schistosomula), *Strongyloides ratti*, *Heligmosomoides polygyrus*, *Necator americanus*, and *Ancylostoma ceylanicum* (all larval stages). These parasites mainly infect humans and other mammals like rodents, dogs, cats, and baboons. In addition, the cytotoxic and antiproliferative potential, respectively, of **1**–**3** was evaluated against PC-3 (human prostate cancer) and HT-29 (human colorectal adenocarcinoma) cell lines.

## 2. Materials and Methods

### 2.1. General Methods

Column chromatography for fractionations or purifications was performed either on silica gel (0.040–0.063 mm, Merck, Germany) or Sephadex LH 20 (Fluka, Steinheim, Germany). Analytical TLCs were performed on pre-coated silica gel F254 aluminum sheets (Merck, Darmstadt, Germany), and spots were detected by their color, their absorbance under a spectrophotometer, or after spraying with vanillin and heating. 

NMR spectra were recorded with an Agilent DD2 400 MHz NMR spectrometer (Varian, Palo Alto, CA, USA) operating at a proton NMR frequency of 400 MHz using a 5-mm inverse detection cryoprobe. 2D NMR spectra were recorded using standard CHEMPACK 8.1 pulse sequences (^1^H,^1^H zTOCSY, ^1^H,^13^C gHSQCAD, ^1^H,^13^C gHMBCAD) implemented in Varian VNMRJ 4.2 spectrometer software (Varian, Palo Alto, CA, USA). The mixing time for the TOCSY experiments was set to 80 msec. The HSQC experiment was optimized for ^1^JCH = 146 Hz with DEPT-like editing and ^13^C-decoupling during the acquisition time. The HMBC experiment was optimized for a long-range coupling of 8 Hz; a two-step ^1^JCH filter was used (130–165 Hz). ^1^H chemical shifts are referenced to internal TMS (^1^H δ = 0 ppm), while ^13^C chemical shifts are referenced to CDCl_3_ (^13^C δ = 77.0 ppm).

The negative ion-electron spray ionization high-resolution mass spectra (ESI-HRMS) were obtained from an API 3200 Triple Quadrupole System (Sciex, Framingham, MA, USA) equipped with a turbo ion spray source, which performs ionization with an ion spray voltage on 70 eV. Sample introduction was performed by direct injection through an Agilent-HPLC 1200 (Agilent, Santa Clara, CA, USA) syringe pump. During the measurement, the mass/charge range from 5 to 1800 was scanned.

### 2.2. Plant Material

The stem bark, leaves, roots, and fruits of *Ozoroa insignis* Del. (Anacardiaceae) were collected in October 2018 in Sehithwa in northwest Botswana. All the samples were authenticated by Mr. Bongani Sethebe from the University of Botswana, where a voucher specimen was deposited with the registration number MD1-10/2018-Fil.

### 2.3. Extract Preparations and Preliminary Anthelmintic Screening

Air-dried plant organ (root bark, stem bark, leaves, and fruits, each 1 g) was extracted by sonication 3 times for 15 min with 10 mL of 80% MeOH at room temperature. The resulting solutions were evaporated to dryness under reduced pressure using a rotary evaporator maintained at 40 °C to afford crude extracts (root bark 122.7 mg, stem bark 98.5 mg, leave 32 mg, and fruits 83.9 mg). From each extract, a stock solution of 1 mg/mL in 4% DMSO was prepared. Each stock sample was screened at the final concentration of 500 µg/mL. Ivermectin (10 μg/mL) was used as a positive control.

### 2.4. Isolation

A total of 86 g of air-dried ground fruits of *Ozoroa insignis* Del. were extracted 3 times by sonication for 15 min with (3 × 400 mL) of 80% MeOH. Filtrates were obtained by using filter papers (Whatman no.1) and were evaporated to dryness under reduced pressure using a rotary evaporator maintained at 40 °C to yeild 28.48 g of crude extract.

This extract, showing 90% anthelmintic activity, was dissolved in 200 mL of water and partitioned between *n*-hexane (500 mL × 3), EtOAc (300 mL × 5), and *n*-butanol (200 mL × 3). The resulting fractions were evaporated to dryness at 40 °C to yield 22.58 g of *n*-hexane extract, 4.16 g of EtOAc extract, 2.73 g of *n*-BuOH extract, and 1.4 g of the remaining aqueous fraction. 

A total of 20.82 g of the most active fraction (*n*-hexane fraction) was adsorbed on an equivalent mass of silica gel and chromatographed over a silica gel column (8 × 36 cm) using *n*-hexane-EtOAc and EtOAc-MeOH gradients as eluent systems. The column was monitored by UV lamp (254 and 366 nm). Fractions of 400–500 mL were collected as follows: [(1–5), *n*-hexane-EtOAc (90:10)], [(6–27), *n*-hexane-EtOAc (8:2)], [(28–35), *n*-hexane-EtOAc (7:3)], [(36–39), *n*-hexane-EtOAc (6:4)], [(40–41), *n*-hexane-EtOAc (1:1)], [(42–43), *n*-hexane-EtOAc (4:6)], [(44), *n*-hexane-EtOAc (3:7)], [(45–46), *n*-hexane-EtOAc (2:8)] [(47–48), *n*-hexane-EtOAc (1:9)] [(49), EtOAc (100%)][(50), EtOAc-MeOH (97.5:2.5)] [(51), EtOAc-MeOH (95:5)] [(52–53), EtOAc-MeOH (92.5:7.5)] [(54–58), EtOAc-MeOH (9:1)] [(59–65), MeOH (100%)]. These fractions were pooled according to their TLC profiles into 5 subfractions F1 to F5 as follows: F1 (1–5), F2 (6), F3 (7), F4 (8–59), and F5 (60–65). Compound **1** precipitated in F4 as a white amorphous solid. 460 mg of F3 was separated by size exclusion chromatography on a Sephadex LH 20 column (1.7 × 40 cm) using DCM-MeOH (7:3) as eluent to afford 35 fractions. Fractions 8–14 were combined to give compound **2** which precipitated as a white amorphous solid. Next, 240 mg of F1 was further separated by Sephadex LH 20 column chromatography (1.7 × 40 cm) using DCM-MeOH (7:3) as eluent. A total of 15 fractions were collected and fractions 4–8 were combined to yield compound **3**.

### 2.5. In Vitro Anthelmintic Bioassay

#### 2.5.1. *Caenorhabditis elegans* Assay 

The Bristol N2 wild-type strain of *Caenorhabditis elegans* was used in the anthelmintic assay. The nematodes were cultured on NGM (Nematode Growth Media) Petri plates using the uracil auxotroph *E. coli* strain OP50 as a food source according to the methods described by Stiernagle [12]. The anthelmintic bioassay was carried out following the method developed by Thomsen et al. [13]. In all the assays, the solvent DMSO (2%) and the standard anthelmintic drug ivermectin (10 μg/mL) were used as negative and positive controls, respectively. All the assays were carried out in triplicate. LC_50_ values were calculated using SigmaPlot 14.0.

#### 2.5.2. Parasitic Helminths

In vitro studies were carried out in accordance with Swiss national and cantonal regulations on animal welfare under permission number 2070 at the Swiss Tropical and Public Health Institute (Swiss TPH). The drug sensitivity assays with *Schistosoma mansoni* [adult and newly transformed schistosomules (NTS)] and *Strongyloides ratti*, *Heligmosomoides polygyrus*, *Necator americanus*, and *Ancylostoma duodenale* were carried out as described in previous publications [14,15] to test the activity of 6-[8(Z)-pentadecenyl] anacardic acid (**1**), 6-[10(Z)-heptadecenyl] anacardic acid (**2**), and 3-[7(Z)-pentadecenyl] phenol (**3**) at 100 µM and 10 µM.

#### 2.5.3. In Vitro Tests on *A. ceylanicum*, *H. polygyrus*, *N. americanus*, and *S. ratti* L3

The life cycles of the assayed nematodes are maintained at the Swiss TPH. *A. ceylanicum*, *H. polygyrus*, and *N. americanus* larvae (L3) were obtained by filtering the feces of infected hamsters (*A. ceylanicum* and *N. americanus*) and mice (*H. polygyrus*) and cultivating the eggs on an agar plate for 8–10 days in the dark at 24 °C. *S. ratti* L3 were acquired as summarized by Garcia and Bruckner [16]. For the drug assay, 30–40 L3 were placed in each well of a 96-well plate for each compound. Larvae were incubated in 175 µL of culture medium with the test drugs at concentrations of 10 and 100 µM. RPMI 1640 (Gibco, Waltham, MA, USA) medium supplemented with 5% amphotericin B (250 µg/mL; Sigma-Aldrich, Buchs, Switzerland) and 1% penicillin 10,000 U/mL (Sigma-Aldrich, Buchs, Switzerland), and streptomycin 10 mg/mL solution (Sigma-Aldrich, Buchs, Switzerland) was used for the assays with *H. polygyrus* L3. Phosphate-buffered saline (PBS; Sigma-Aldrich, Buchs, Switzerland) supplemented with 1% penicillin (10,000 U/mL) and streptomycin (10 mg/mL) solution was used to incubate *S. ratti* L3. *Ancylostoma ceylanicum* and *N. americanus* L3 stages were incubated in Hanks’ balanced salt solution (HBSS; Gibco, Waltham, MA, USA) supplemented with 10% amphotericin B and 1% penicillin (10,000 U/mL) and streptomycin (10 mg/mL) solution. Larvae were kept in the dark at room temperature for 72 h, after which the drug effect was evaluated. For this, the total number of L3 per well was determined. Then, 50–80 µL of hot water (≈80°C) was added to each well, and the larvae that responded (the moving worms) were counted. The proportion of larval death was determined.

#### 2.5.4. In Vitro Tests on *S. mansoni*

The in vitro tests on *S. mansoni* were carried out as described in literature [14]. Briefly, to obtain newly transformed schistosomula (NTS), cercariae were collected from infected *Biomphalaria glabrata* snails (maintained at Swiss TPH) and were mechanically transformed. The NTS were kept in the incubator (37 °C and 5% CO_2_) in medium 199 (Gibco, Waltham, MA, USA), supplemented with 5% fetal calf serum (FCS; Bioconcept, Allschwil, Switzerland) and 1% penicillin/streptomycin and 1% (*v*/*v*) antibacterial/antifungal solution until usage. Adult *S. mansoni* worms were collected by dissecting the mesenteric veins of infected mice at day 49 post-infection. For NTS and adult *S. mansoni*, transparent flat-bottom 96- and 24-well plates were used, respectively (Sarstedt, Nürmbrecht, Germany). 30–40 NTS were incubated with the test drug (1, 10, and 100 μM) in 250 μL of M199 medium (Gibco, Waltham, MA, USA) supplemented with 5% (*v*/*v*) FCS (Bioconcept, Allschwil, Switzerland), 1% (*v*/*v*) penicillin/streptomycin solution (Sigma-Aldrich, Bruch, Switzerland) for up to 72 h at 37 °C and 5% CO_2_. The experiment was conducted in triplicate. For the adult *S. mansoni* assay, at least 3 worms (both sexes) were incubated in a final volume of 2 mL RPMI 1640 supplemented with 5% (*v*/*v*) FCS and 1% (*v*/*v*) penicillin/streptomycin at 37 °C and 5% CO_2_ for 72 h and the test drug at 10 and 100 μM. The experiment was conducted in duplicate. NTS and adult worms were judged via microscopic readout 72 h after incubation; they were scored according to motility, morphology, and granularity (scores from 0 to 3).

### 2.6. Cytotoxic Effects on Human Cancer Cell Lines

The investigated cell lines, PC-3 (human prostate adenocarcinoma) and HT-29 (human colorectal adenocarcinoma), were purchased from ATCC (Manassas, VA, USA). The cell culture medium RPMI 1640, the supplements FCS and L-glutamine, as well as PBS and trypsin/EDTA, were purchased from Capricorn Scientific (Ebsdorfergrund, Germany). Culture flasks, multi-well plates, and further cell culture plastics were from Greiner Bio-One (Frickenhausen, Germany) and TPP (Trasadingen, Switzerland), respectively. Resazurin used for the cell viability assays was purchased from Sigma-Aldrich (Taufkirchen, Germany). 

#### 2.6.1. Cell Culture

The compounds of interest were studied for their cytotoxic and cytostatic, respectively, impact on 2 human cancer cell lines, PC-3 (prostate adenocarcinoma) and HT-29 (colorectal adenocarcinoma). Both cell lines were cultured in RPMI 1640 medium supplemented with 10% heat-inactivated FCS, 2 mM L-glutamine, and 1% penicillin/streptomycin, in a humidified atmosphere with 5% CO_2_ at 37 °C. Routinely, cells were cultured in T-75 flasks until reaching subconfluency (~80%). Subsequently, cells were harvested by washing with PBS and detached by using trypsin/EDTA (0.05% in PBS) prior to cell passaging and seeding for sub-culturing and assays in 96-well plates, respectively [17]. 

#### 2.6.2. In Vitro Cell Viability Assay

The antiproliferative, i.e., cytotoxic or cytostatic effect of the compounds **1**, **2,** and **3,** was investigated by using a fluorometric resazurin-based cell viability assay as described previously [18]. For that purpose, cancer cells were seeded in low density in 96-well plates −6000 cells/100µL/well in case of PC-3 cells, −10,000 cells/100µL/well in case of HT-29 cells. Subsequently, the cells were allowed to adhere overnight, followed by a 48 h treatment with the compounds of interest. For that purpose, dilution series (12.5, 25, 50, and 100 µM) of the compounds **1**, **2,** and **3** were prepared in standard culture medium, starting from 20 mM DMSO stock solutions. For control measures, cells were treated in parallel with 0.5% DMSO (negative control, representing the final DMSO content of the highest, 100 µM test concentration), and 100 µM digitonin (positive control, for data normalization, set equal to 0% cell viability), both in standard growth medium. As soon as the 48 h incubation was finished, cells were treated with a final resazurin concentration of 50 µM (based on 2.5 mM aqua bidest. stock) for 4 h under standard growth conditions. During that time, just viable, metabolically active cells were able to convert resazurin to its reduced and fluorescent derivative resorufin that was measured (λ_exc_ = 540 nm, λ_em_ = 590 nm) by using a SpectraMax M5 multi-well plate reader (Molecular Devices, San Jose, CA, USA). Cell viability data were determined with technical quadruplicates in biological triplicates. GraphPad Prism 8 (GraphPad Software, San Diego, CA, USA), Statsdirect software version 3.2.8 (Statsdirect, Wirral, UK), and Microsoft Excel 2013 (Microsoft, Redmond, WA, USA) were used for data analyses.

## 3. Results

**Figure 1:**
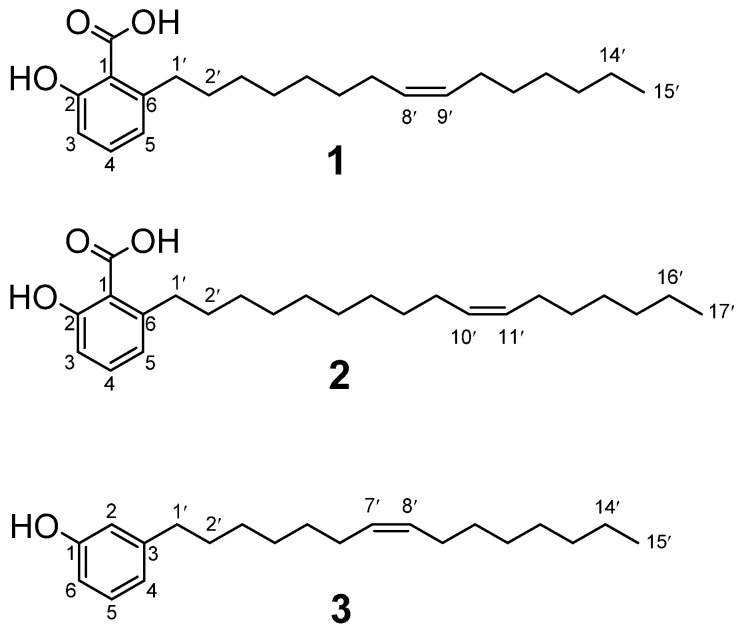
Structures of isolated compounds **1**–**3**.

The anthelmintic activity of 80% methanol crude extracts of stem bark, leaves, roots, and fruits of *O. insignis* was evaluated using *C. elegans* as a model organism and revealed that the extract of fruits was the most active with mortality activity of 91.73 ± 6.05% compared to the extracts of roots, leaves and stem bark which had 35.11 ± 2.91%, 17.58 ± 3.28% and 16.42 ± 7.86% percentage mortality, respectively, at a concentration of 500 µg/mL (Table S1; Supplementary Materials). After partitioning of the fruit extract between water and different organic solvents, it was observed that the *n*-hexane fraction was the most active with 92.54 ± 0.81% mortality at a concentration of 500 µg/mL. Other fractions from partitioning had activity below 5% as follows: EtOAc (4.54 ± 4.01), *n*-butanol (4.28 ± 1.07), and the remaining aqueous fraction (4.90 ± 1.70) (Table S2; Supplementary Materials). The *n*-hexane fraction was subjected to column chromatography on silica gel using *n*-hexan /ethyl acetate (increasing polarity) as solvents. Using bioassay-guided fractionation, compound **1** (Figure 1) was isolated as a white amorphous solid and identified as 6-[8*(Z)*-pentadecenyl] anacardic acid (**1**) based on its spectral data, mainly HRMS, 1D, and 2D NMR (Figures S1–S9, Tables S3–S4; Supplementary Materials), and by comparison with detailed reported data [3,19]. Compound **2** (Figure 1) was isolated as a white amorphous solid and identified as 6-[10(*Z*)-heptadecenyl] anacardic acid (**2**) by comparing its spectral data (Figures S10–S18, Tables S3 and S4; Supplementary Materials) with that reported in literature [20,21,22]. Compound **3** (Figure 1) was isolated as a clear oily liquid and determined to be 3-[7(*Z*)-pentadecenyl] phenol (**3**) based on detailed HR-MS, 1D, and 2D NMR studies (Figures S19–S28, Tables S3–S4; Supplementary Materials) and comparison with reported spectral data [23].

The isolated pure compounds **1** and **2** were re-tested against *C. elegans* and exhibited 100% activity at the tested concentration of 500 µg/mL. The LC_50_ value of **1** was determined to be 51.9 µM, while the LC_50_ of **2** was determined to be 93.4 µM (Tables S5 and S6; Figure S29; Supplementary Materials) (positive control ivermectin 10 μg/mL). Re-testing of **3** and salicylic acid (data not shown) exhibited no anthelmintic activity against *C. elegans*. 

Compounds **1**–**3** were next tested against 5 parasitic helminths *Schistosoma mansoni* (adult and newly transformed schistosomules (NTS)), and the larval stages of *Strongyloides ratti*, *Heligmosomoides polygyrus*, *Necator americanus*, and *Ancylostoma ceylanicum*. The compounds were tested at 2 concentrations, 100 µM and 10 µM (Table 1).

Compounds **1**, **2,** and **3** showed good activity against adult *S. mansoni* and newly transformed schistosomula, killing 100% of the organisms at 100 µM. At the same test concentration of 100 µM, 76.3% of *S. ratti* were killed by compound **1,** while compounds **2** and **3** had a mortality rate of less than 30%. All 3 compounds showed weak activity against *N. americanus* at 100 µM, killing only 26.7% to 35.8% of the larvae. The activity of **1**–**3** against *H. polygyrus* was weak at 100 µM, with less than 30% of the larvae dying. Compounds **1** and **3** showed moderate activity against *A. ceylanicum*, killing 50.9% and 46.1% of the parasites, respectively, at 100 µM, while compound **2** killed only 12.6% of the larvae at the same concentration.

At the reduced test concentration of 10 µM, compounds **1** and **2** showed weak anthelmintic activity against S. *ratti, H. polygyrus*, *N. americanus*, and *A. ceylanicum*, killing less than 30% of the larvae. Also, at 10 µM, compounds **1** and **2** had reduced activity against adult *S. mansoni* and NTS (<30% activity). Compound **3** showed a high activity of 95% against NTS at 10 µM. Compound **3** also showed moderate anthelmintic activity against *A. ceylanicum*, killing 41% of the organisms at the test concentration of 10 µM. Testing at a further reduced concentration of 1 µM against NTS resulted in a mortality rate of 50% for **3**, while compounds **1** and **2** were inactive. 

**Figure 2:**
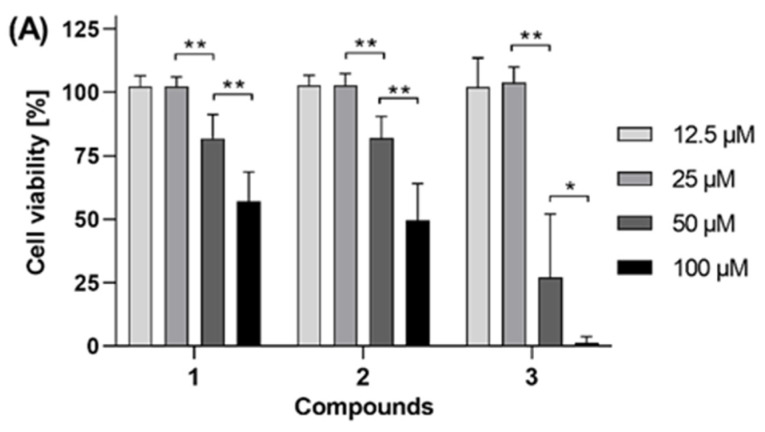

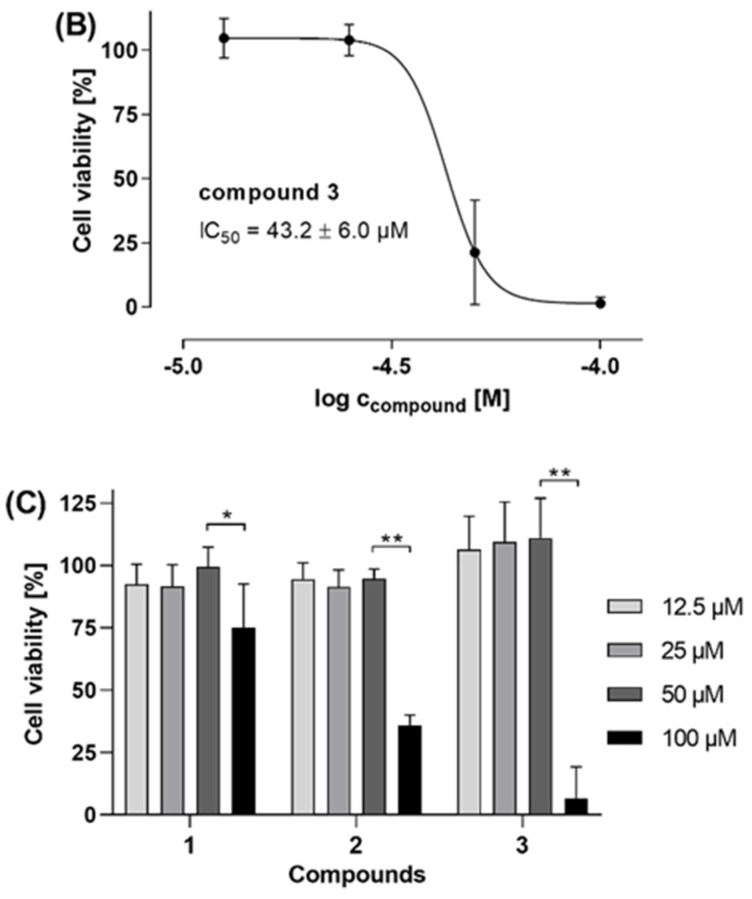
Effects of compounds **1**, **2,** and **3** on the viability of (**A**) PC-3 (human prostate cancer) and (**C**) HT-29 (human colorectal cancer) cells, respectively. The IC_50_ curve of compound **3** in PC-3 cells is shown in (**B**). After 48 h of compound treatment, the cell viability was measured by using a fluorometric resazurin-based assay. Data represent biological triplicates, each with technical quatruplicates. Digitonin (100 µM) was used as positive control compromising the cells to yield 0% cell viability after 48 h. Data were analyzed by using GraphPad Prism 8. Statistical significances were evaluated by using Brown-Forsythe and Welch one-way ANOVA tests, including Tamhane’s multiple comparison test as well as ordinary one-way ANOVA including Sidak’s multiple comparison test; in all cases with ** *p* < 0.0001, and * *p* < 0.05.

Compounds **1**, **2,** and **3** were tested for their effects on the viability of two human cancer cell lines, namely PC-3 prostate adenocarcinoma cells and HT-29 colorectal adenocarcinoma cells (Figure 2). The in vitro cell viability and cytotoxicity assays were measured by using a fluorometric resazurin-based read-out after 48 h cell treatment. The saponin digitonin (100 µM), a very potent permeabilizer of cell membranes, was used as positive control compromising the cells to yield 0% cell viability after 48 h. The compounds were tested with at least 4 concentrations, i.e., 12.5 µM, 25 µM, 50 µM, and 100 µM.

## 4. Discussion

The free-living nematode *C. elegans* is a good model organism due to its ease of laboratory maintenance, and it has enough similarities to the parasitic worms to act as an approximate test model [24]. For example, the animal parasitic suborder Strongylida including the human hookworms *Ancylostoma* and *Necator*, is closely related to *C. elegans* and, therefore, the latter is an excellent model organism for these pathogens [25]. The bioassay-guided fractionation of the fruit extract of *O. insignis* showed that the activity against *C. elegans* was found in the *n*-hexane fraction while the ethyl acetate, *n*-butanol, and aqueous fractions have much lower activity against *C. elegans.* Compound **3** showed low activity against *C. elegans* but moderate to strong activity against some of the parasitic helminths, e.g., *Schistosoma* and NTS. This demonstrates that although *C. elegans* is suitable for bioassay-guided isolation, there may be compounds that are weakly active against *C. elegans* but may be more active against parasitic helminths. This may be due to *C. elegans* innate physical and enzymatic defenses to xenobiotics, which are factors important for its survival in the natural environment. This makes it difficult for some chemicals to access the worm and the compounds and, therefore, require high concentrations to bring about observable changes in phenotype [26]. 

Compounds **1** and **2** showed strong anthelmintic activity against *C. elegans*, while compound **3** showed no activity. The low activity of compound **3** against *C. elegans* may have been due to the absence of the carboxylic acid functional group, which was absent in compound **3** but present in both compounds **1** and **2**. When *C. elegans* was tested against salicylic acid (data not shown), this substance showed no activity against the free-living worm, giving an indication that the alkyl chain in compounds **1** and **2,** which is absent in salicylic acid, is necessary for the anthelmintic activity against *C. elegans*. Remarkably, compound **3** showed better activity against NTS and *A. ceylanicum* than compounds **1** and **2**. 

The strong anthelmintic activity of compounds **1**, **2,** and **3** against *S. mansoni* and NTS gives evidence to the reported use of *O. insignis* roots in the treatment of schistosomiasis [3]. The strong activity of 6-[8(*Z*)-pentadecenyl] anacardic acid (**1**) is in accordance with data published by Wang et al. [27] on the activity of ginkgolic acids C13:0 and C15:1 on *Pseudodactylogyrus*, a parasite of the gills of aquacultured European eels. 6-[10(*Z*)-heptadecenyl] anacardic acid (**2**) has been shown to be toxic against the citrus red mite *Panonychus citri* by Pan et al. [28]. To the best of our knowledge, there are no reports on the anthelmintic activity of 3-[7(*Z*)-pentadecenyl] phenol (**3**), but the compound has shown activity against the brine shrimp *Artemia salina* [23]. This study provides scientific evidence of the anthelmintic activity of *O. insignis* fruits extract, which was far more active than root, stem bark, and leaf extracts. 

As shown in Figure 2A,C, compounds **2** and **3** showed antiproliferative activity in both PC-3 human prostate adenocarcinoma cells and HT-29 human colorectal adenocarcinoma cells, while compound **1** exhibited antiproliferative activity only on PC-3 cells. Whereas 100 µM of both compound **1** and **2** reduced the viability of PC-3 cells by approximately 50% (Figure 2A), compound **3** showed enhanced antiproliferative activity against PC-3 cells with a calculated IC_50_ value of 43.2 ± 6.0 µM (Figure 2B). In HT-29 cells (Figure 2C), compound **3** was less active, with an estimated IC_50_ between 50 and 100 µM. In the same cells, the IC_50_ of compound **2** is estimated to be just below 100 µM, since this concentration of compound **2** reduced the HT-29 cell viability by more than 50% (down to 36% viability). Contrarily, compound **1** did not cause a 50% reduction (IC_50_) of HT-29 viability even with a 100 µM concentration. Taken together, compound **3** is the most active of the tested compounds, permitting an antiproliferative effect with an IC_50_ value of 43.2 µM in human PC-3 prostate adenocarcinoma cells. Furthermore, since in Figure 2B, the lower curve plateau reached 0% cell viability, it can be stated that the antiproliferative effect is caused by cytotoxicity of compound **3**, i.e., induction of cell death, not just cytostatic cell growth arrest. For compound **1**, these findings are in accordance with data published by Rea et al. [4] on the bioactivity-directed (cytotoxicity) chromatographic separation and isolation of compounds named anacardic acid and ginkgoic acid (= 6-[8(*Z*)-pentadecenyl] anacardic acid, **1**), the main active constituents of *O. insignis* bark extract. The authors determined the IC_50_ values against the human cell lines Hep-G2 (human hepatocellular carcinoma) as 385 ± 71 µM, MDA-MB-231 (human mammary adenocarcinoma) as 289 ± 54 µM, Hs 578T (human mammary ductal carcinoma) as 88.9 ± 8.7 µM, MCF-7 (human mammary adenocarcinoma) as >300 µM, SK-MEL-28 (human melanoma) 199 ± 11 µM, and 5637 (human primary bladder carcinoma) as 131.3 ± 1.0 µM. However, Rea et al. [4] did not obtain compounds **2** and **3** from *O. insignis* bark, either through bioactivity-directed (*Artemia salina* lethality) fractionation or bioactivity-directed (cytotoxicity) chromatographic separation as we could demonstrate here for the fruits of *O. insignis*. Itokawa and coauthors [20] also reported compound **1** had potent activity against Sarcoma 180 ascites in mice using the total packed cell volume method (grow ratio 17.4%).

Compounds **1**–**3** are also constituents of *G. biloba*, the most valued and ancient among medicinal plants [29]. The growth inhibitory effects of **1**–**3** isolated from *G. biloba* were examined on several human cancer cell lines and a normal cell line. All compounds inhibited the growth of human cancer cells such as HCT-15 (colon), MCF-7 (breast), A-549 (lung), HT-1197 (bladder), and SKOV-3 (ovary). Interestingly, compounds **1** and **3** were less cytotoxic on the normal colon cell line (CCD-18-Co) than on the corresponding colon carcinoma (HCT-15). Unfortunately, data for compound **2** on CCD-18-Co are not presented [30]. Very recently, it was reported that compound **1** inhibited cell proliferation, migration, epithelial-mesenchymal transition, and overall protein SUMOylation in BGC823 and HGC27 cells. Additionally, **1** hindered the progression of gastric cancer by inhibiting the SUMOylation of IGF-1R [31]. 

In general, compounds **1** and **2** possess a wide range of bioactive properties and can exert diverse pharmacological activities [23,32]. Compound **1** has been shown to inhibit HIV protease activity in a cell-free system and HIV infection in PBMCs without significant cytotoxicity [33] and acts as a multi-target inhibitor of key enzymes in pro-inflammatory lipid mediator biosynthesis [34]. Compound **1** has also shown strong antibacterial activity against gram-positive bacteria [35]. Very recently, it was demonstrated that compounds **1** and **2** exhibited strong SARS-CoV-2 3Clpro inhibition with IC_50_’s of 3.45 ± 0.78 µM and 1.19 ± 0.15 µM, respectively [36]. Compound **1** also inhibited SARS-CoV-2 Papain-like protease and 3C-like protease with IC50s of ca. 16.3 μM and 1.79 μM, respectively, in vitro in a dose-dependent manner. The compound also blocked SARS-CoV-2 replication with an EC_50_ of ca. 8.3 μM in Vero E6 cells [37].

## 5. Conclusions

The present study aimed to assess the anthelmintic potential of different organs of *O. insignis*, a medicinal plant used in traditional folk medicine for the treatment of intestinal worms. The results obtained demonstrated for the first time the in vitro anthelmintic potential of the fruits extract, which had the best activity compared to stem bark, leaves, and roots. To recognize the active principles in the fruits, we isolated using bioassay-guided fractionation 3 known compounds and evaluated their anthelmintic properties against a panel of parasites mainly infecting humans and/or other mammals like rodents, dogs, cats, and baboons. The evaluation of the anthelmintic properties revealed strong antiparasitic activity of all the isolated compounds (**1**–**3**) against *S. mansoni* and could justify the use of *O. insignis* roots in the treatment of schistosomiasis. In addition, the cytotoxic and antiproliferative potential of the compounds was evaluated against PC-3 (human prostate cancer) and HT-29 (human colorectal adenocarcinoma) cell lines, respectively. Compound **3** was the most active, showing antiproliferative activity against PC-3 cell lines. In summary, the anthelmintic activities, cytotoxic and antiproliferative potential of compounds **1**–**3** are promising, and this study gives evidence that *O. insignis* fruit extracts deserve further studies in order to fully investigate the potential of discovering new anthelmintics and anticancer drugs. So far, the pharmacodynamics as well as the pharmacokinetic parameters are unknown and should be investigated. Moreover, expanded toxicological investigations should be performed to validate the safety of the compounds.

## Figures and Tables

**Table 1:** Anthelmintic activity against parasitic organisms.

Organism	1 Activity% *	2 Activity% *	3 Activity% *
NTS ** (100 µM)	100 ± 0 ***	100 ± 0 ***	100 ± 0 ***
NTS ** (10 µM)	14.0 ± 2	30.0 ± 3.3	95.0 ± 5 ***
NTS ** (1 µM)	0	0	50 ± 0 ***
*S. mansoni* adults (100 µM)	100 ± 0 ***	100 ± 0 ***	100 ± 0 ***
*S. mansoni* adults (10 µM)	25.3 ± 0	0	25.0 ± 0
*S. ratti* L3 (100 µM)	76.3 ± 0.4 ***	19.7 ± 4.8	23.8 ± 8.3
*S. ratti* L3 (10 µM)	7.0 ± 11.9	2.2 ± 4.3	16.4 ± 8.1
*N. americanus* L3 (100 µM)	27.2 ± 12.8	35.8 ± 6.9	26.7 ± 0.1
*N. americanus* L3 (10 µM)	14.4 ± 3.4	28.7 ± 2.1	21.2 ± 5.3
*H. polygyrus* L3 (100 µM)	21.6 ± 17.6	25.9 ± 3.1	21.4 ± 0.7
*H. polygyrus* L3 (10 µM)	1.8 ± 7.2	24.6 ± 0.4	8.7 ± 3.3
*A. ceylanicum* L3 (100 µM)	50.9 ± 6.1 ***	12.6 ± 2.5	46.1 ± 7.4 ***
*A. ceylanicum* L3 (10 µM)	12.2 ± 3.2	12.3 ± 5.6	41 ± 10.4

* mortality % based on 3 replicates; ** NTS = newly transformed schistosomula; *** compound activities determined to be significant using Kruskal–Wallis non-parametric test (Statsdirect software version 3.2.8) with *p* < 0.05.

## Supplementary Materials

The following are available online at https://www.mdpi.com/article/10.3390/biom11121893/s1, Figures S1–S9: HRMS, 1D and 2D NMR of compound **1**; Figures S10–S18: HRMS, 1D and 2D NMR of compound **2**; Figures S19–S27: HRMS 1D and 2D NMR of compound **3**; Figure S28: Important fragment ions on the TOF MS2 spectra of compounds **1**–**3**; Tables S1 and S2: Anthelmintic activity of extracts and fractions (testorganism *C. elegans).* Table S3: ^1^H NMR data (400 MHz, CDCl_3_, δ in ppm) of 6-[8(*Z*)-pentadecenyl] anacardic acid (**1**), 6-[10(*Z*)-heptadecenyl] anacardic acid (**2**) and 3-[7(*Z*)-pentadecenyl] phenol (**3**) including HMBC (H to C); Table S4: ^13^C NMR data (100 MHz, CDCl_3_, δ in ppm) of 6-[8(*Z*)-pentadecenyl] anacardic acid (**1**), 6-[10(*Z*)-heptadecenyl] anacardic acid (**2**) and 3-[7(*Z*)-pentadecenyl] phenol (**3**); Tables S5 and S6: Anthelmintic activity of **1**–**3** (testorganism *C. elegans).* Figure S29: Graph for LC_50_ of 6-[8(*Z*)-pentadecenyl] anacardic acid (**1**) and 6-[10(*Z*)-heptadecenyl] anacardic (**2**) (testorganism *C. elegans).*
