# Supplementary material for: Anthelmintic Activity and Cytotoxic Effects of Compounds Isolated from the Fruits of Ozoroa insignis Del. (Anacardiaceae)
Source: Biomolecules. 2021 Dec 17;11(12):1893. doi: 10.3390/biom11121893 (PMC8699734; doi:10.3390/biom11121893)
Supplement: Supplementary file 1 [file biomolecules-11-01893-s001.zip › biomolecules-1478492-supplementary.pdf]

Supplementary Material

Anthelmintic activity and cytotoxic effects of compounds isolated from the fruits of *Ozoroa insignis* Del. (Anacardiaceae)

Mthandazo Dube[1], Mohamad Saoud[1], Robert Rennert[1], Ghislain Wabo Fotso[2], Kerstin Andrae-Marobela[3], Peter Imming[4], Cécile Häberli[5,6], Jennifer Keiser[5,6], Norbert Arnold[1*]

[1] Department of Bioorganic Chemistry, Leibniz Institute of Plant Biochemistry, Weinberg 3, D-06120 Halle (Saale), Germany
[2] Department of Organic Chemistry, Faculty of Science, University of Yaoundé 1, P.O. Box 812, Yaoundé, Cameroon
[3] Department of Biological Sciences, Faculty of Science, University of Botswana, P.O. Box 0022, Gaborone, Botswana
[4] Institute of Pharmacy, Faculty of Natural Sciences, Martin-Luther-University Halle-Wittenberg, D-06120 Halle (Saale), Germany
[5] Helminth Drug Development Unit, Department of Medical Parasitology and Infection Biology, Swiss Tropical and Public Health Institute, Socinstr. 57, CH-4051 Basel, Switzerland
[6] University of Basel, CH-4051 Basel, Switzerland
*Correspondence: Norbert.Arnold@ipb-halle.de; Tel.: +49-345-5582-1310 (N.A.)

<u>Figures S1 - S9: HR-ESI-MS, 1D and 2D NMR of 6-[8(*Z*)-pentadecenyl] anacardic acid (1)</u>

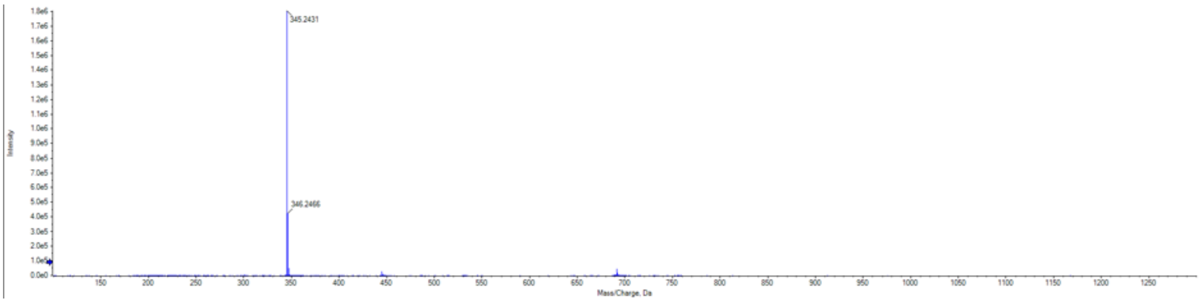

Figure S1: HR-ESI-MS spectrum (neg. mode) of 6-[8(Z)-pentadecenyl] anacardic acid (**1**).

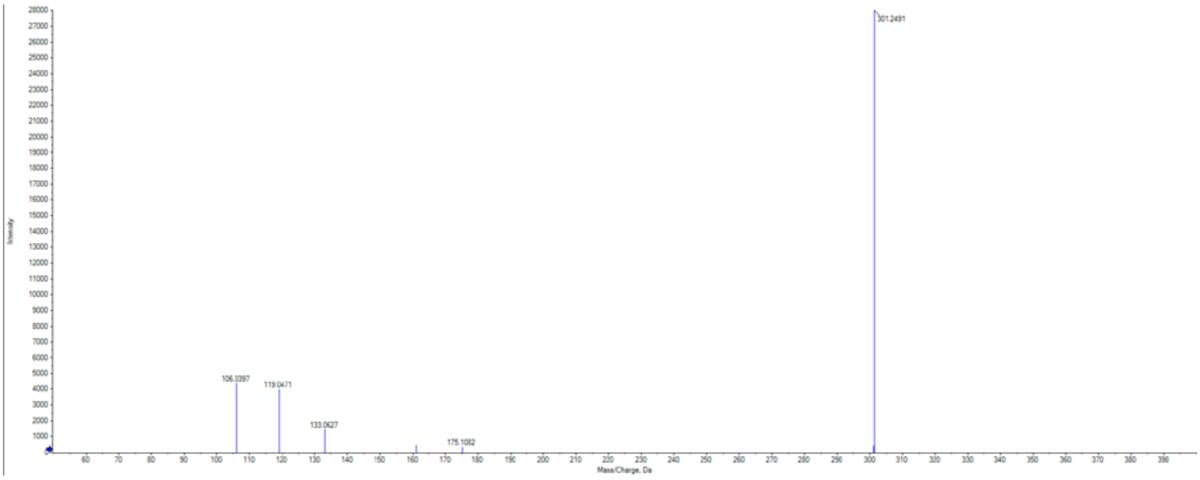

Figure S2: TOF MS^2 (neg. mode) of 6-[8(*Z*)-pentadecenyl] anacardic acid (**1**).

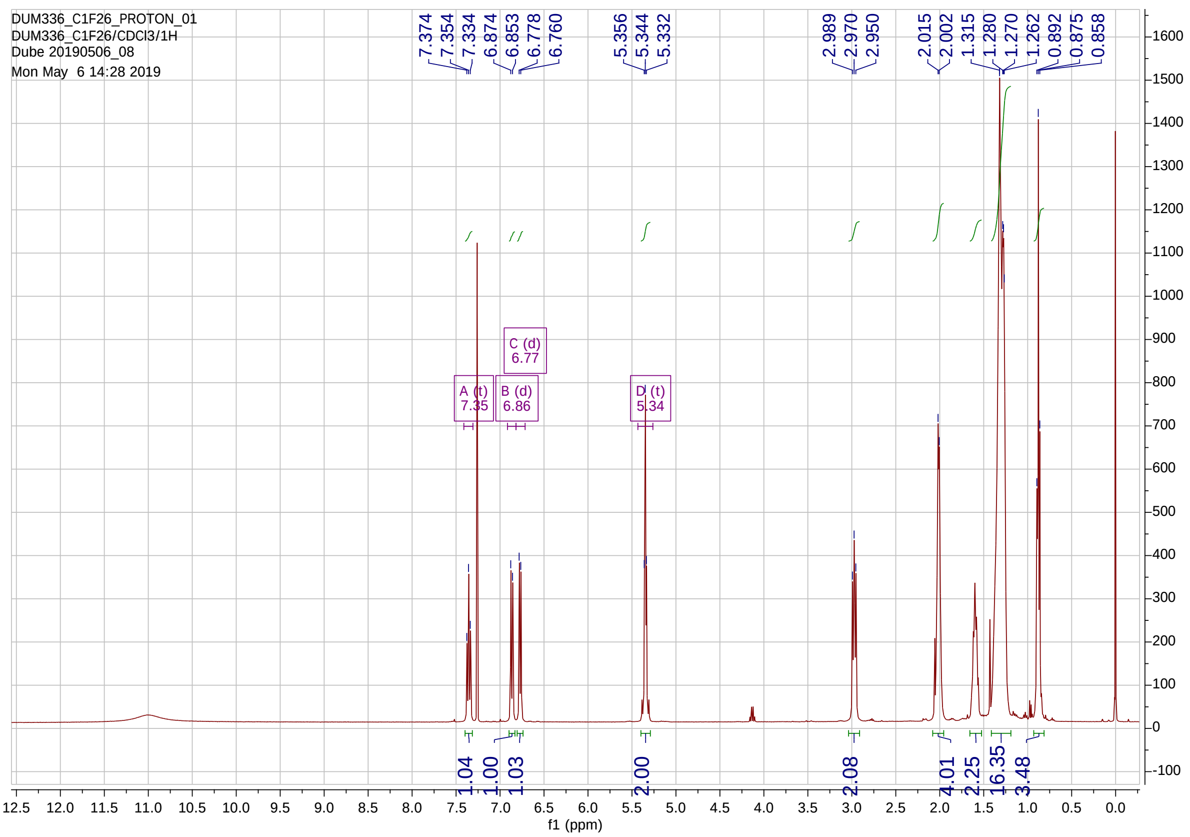

Figure S3: ^{1}H NMR spectrum of 6-[8(Z)-pentadecenyl] anacardic acid (**1**) in $CDCl_3$ (400 MHz).

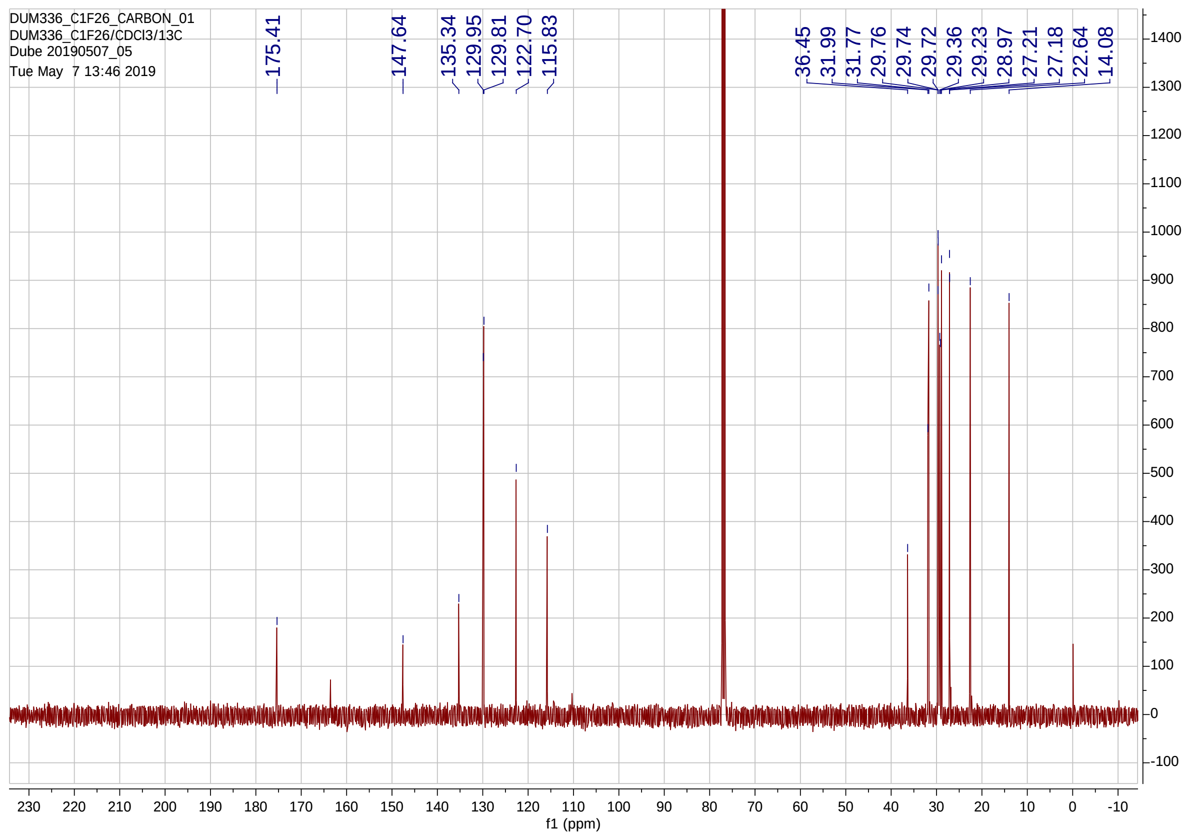

Figure S4: ^{13}C NMR spectrum of 6-[8(Z)-pentadecenyl] anacardic acid (**1**) in $CDCl_3$ (400 MHz).

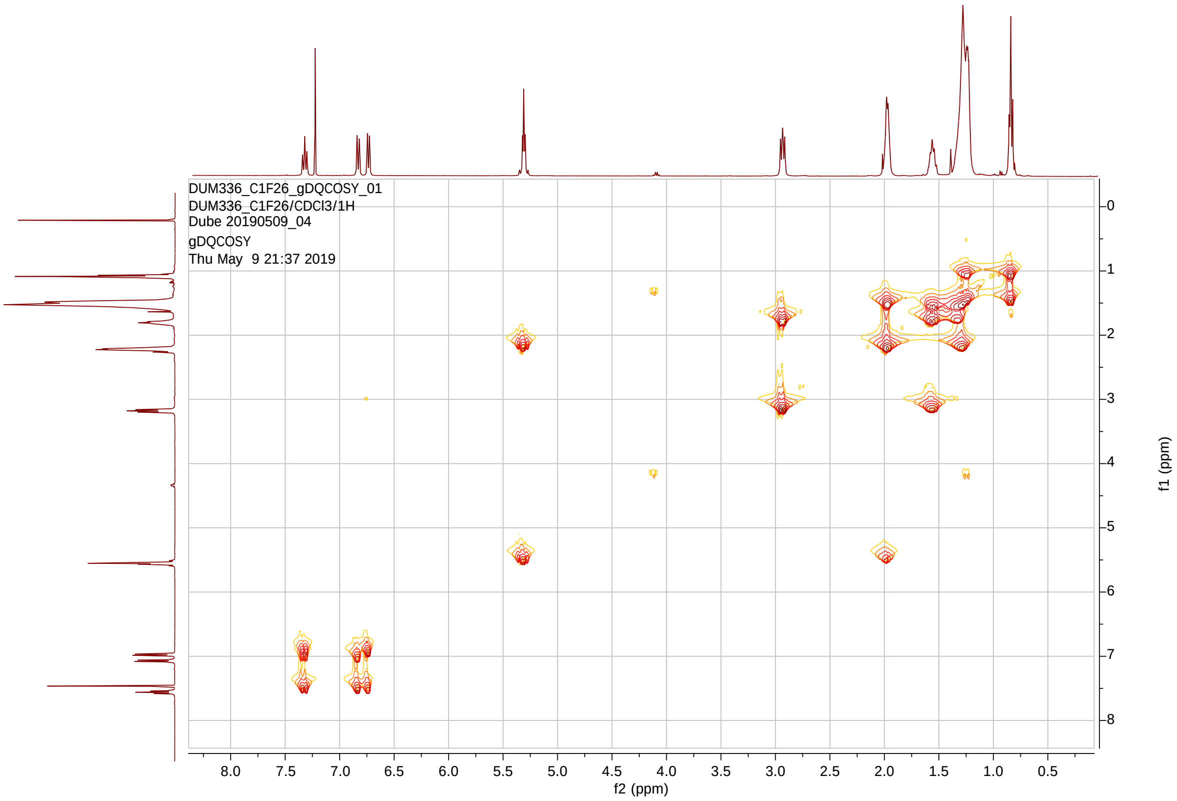

Figure S5: gDQCOSY spectrum of 6-[8(Z)-pentadecenyl] anacardic acid (**1**) in $CDCl_3$ (400 MHz).

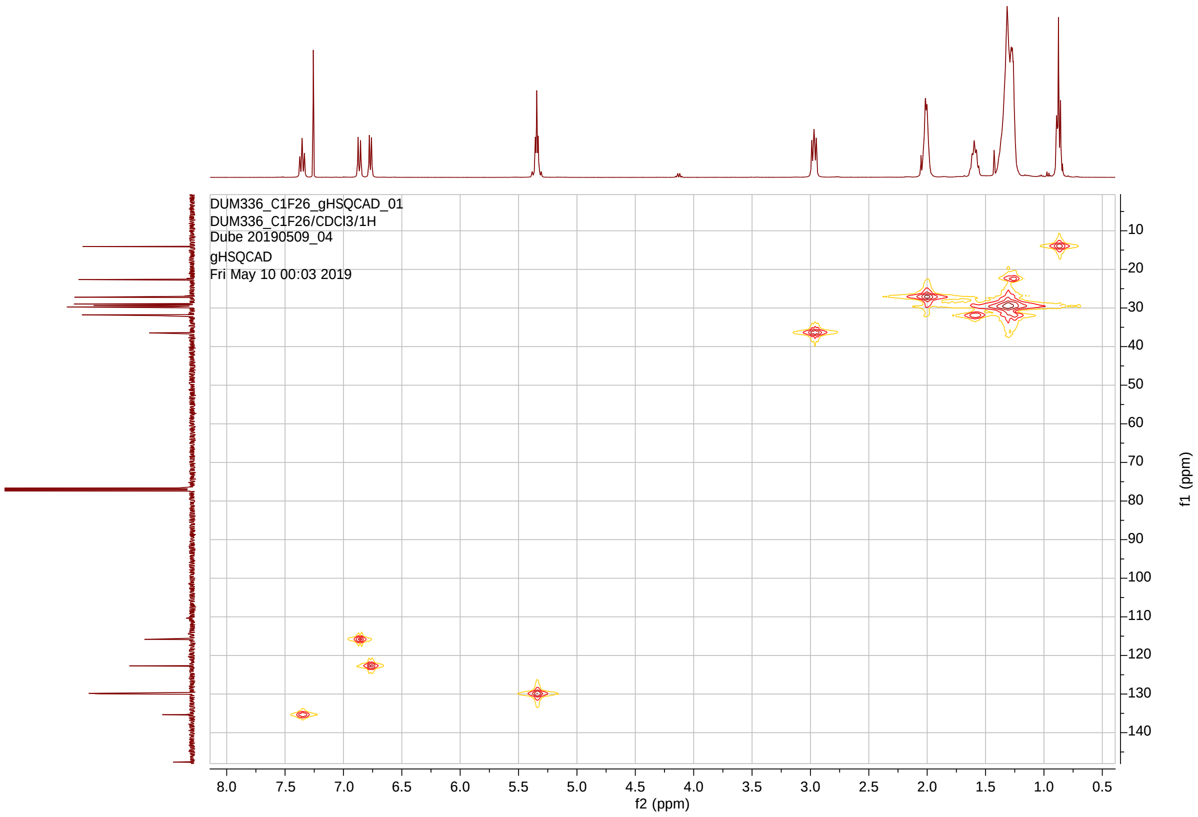

Figure S6: gHSQC spectrum of 6-[8(Z)-pentadecenyl] anacardic acid (**1**) in $CDCl_3$ (400 MHz).

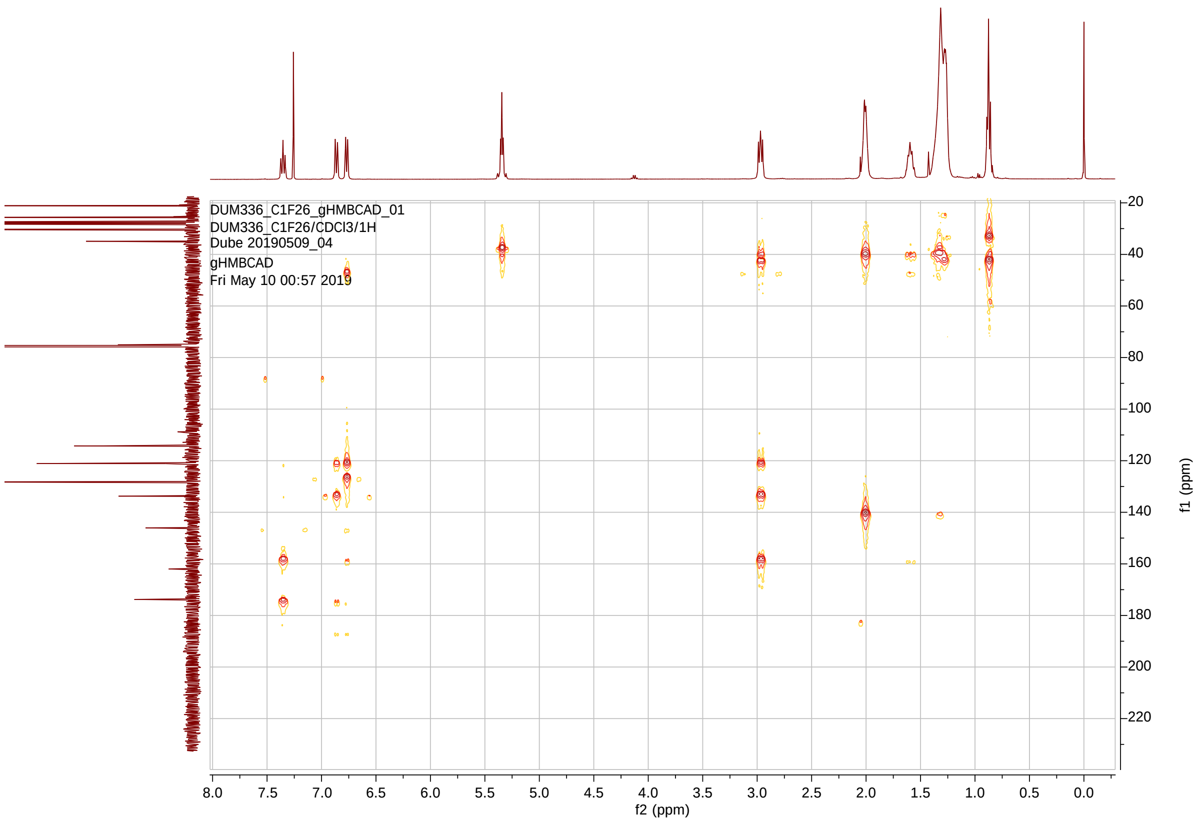

Figure S7: gHMBC spectrum of 6-[8(Z)-pentadecenyl] anacardic acid (**1**) in $CDCl_3$ (400 MHz).

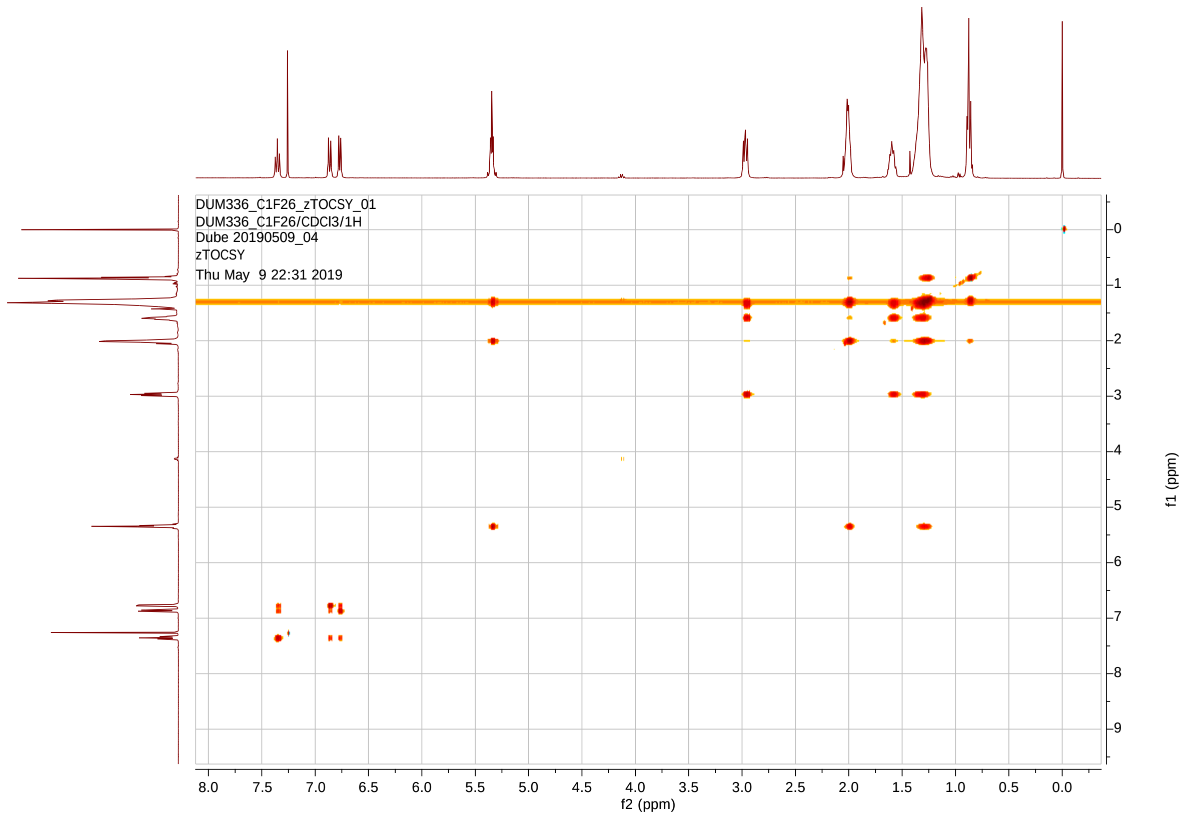

Figure S8: gTOCSY spectrum of 6-[8(Z)-pentadecenyl] anacardic acid (**1**) in $CDCl_3$ (400 MHz).

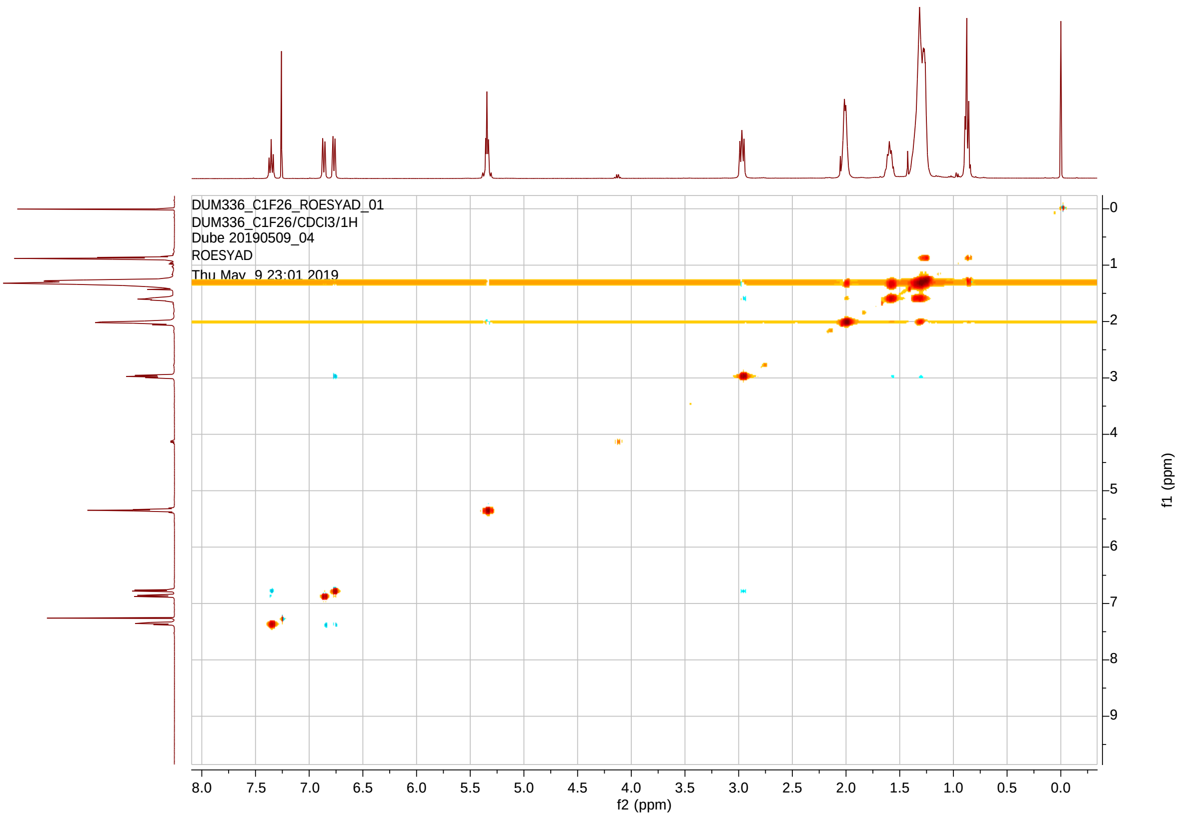

Figure S9: gROESY spectrum of 6-[8(Z)-pentadecenyl] anacardic acid (**1**) in $CDCl_3$ (400 MHz).

<u>Figures S10-S18: HR-ESI-MS, 1D and 2D NMR of 6-[10(*Z*)-heptadecenyl] anacardic acid (2)</u>

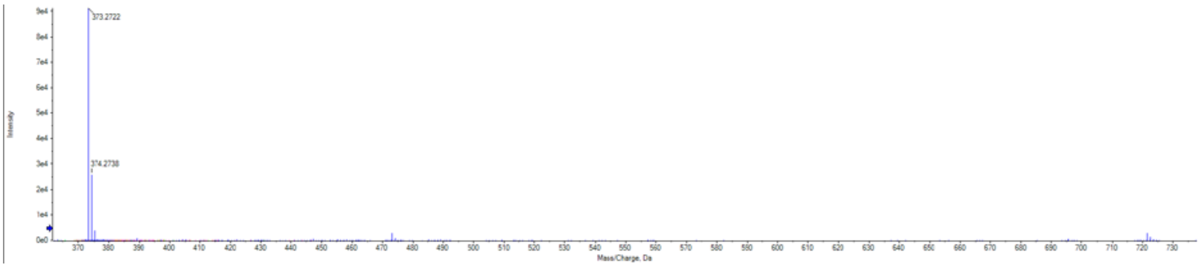

Figure S10: HR-ESI-MS spectrum (neg. mode) of 6-[10(*Z*)-heptadecenyl] anacardic acid (**2**).

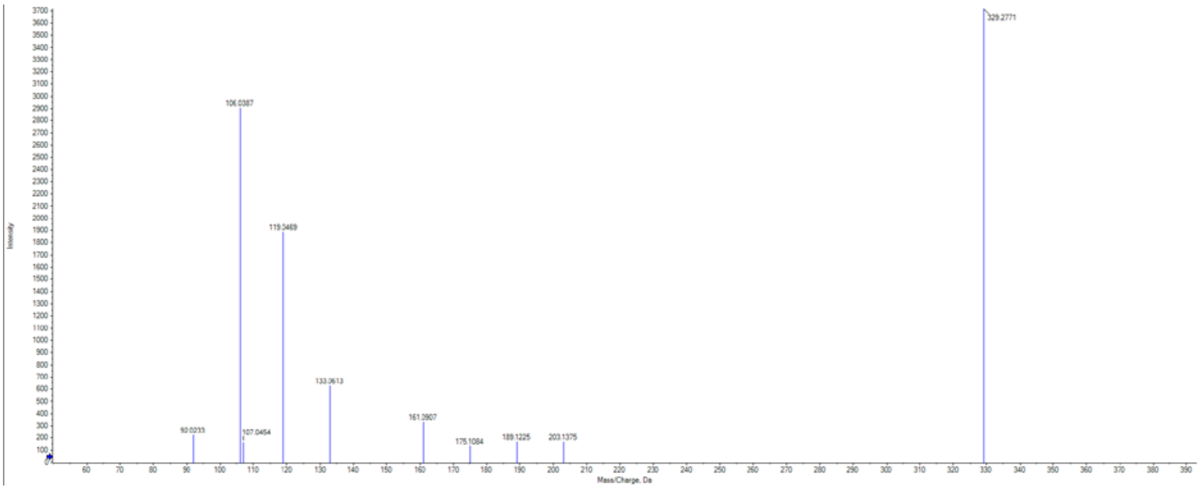

Figure S11: TOF MS² spectrum (neg. mode) of 6-[10(*Z*)-heptadecenyl] anacardic acid (**2**).

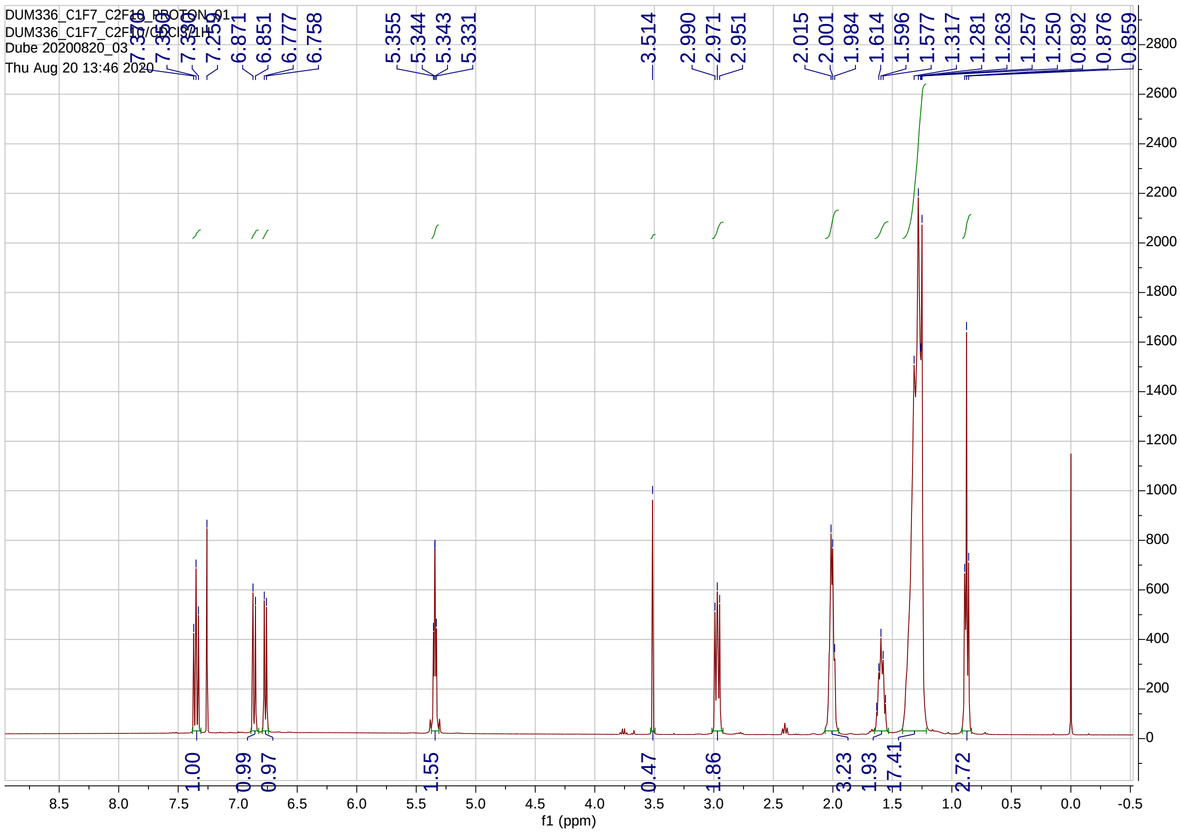

Figure S12: ¹H NMR spectrum of 6-[10(Z)-heptadecenyl] anacardic acid (**2**) in $CDCl_3$ (400 MHz).

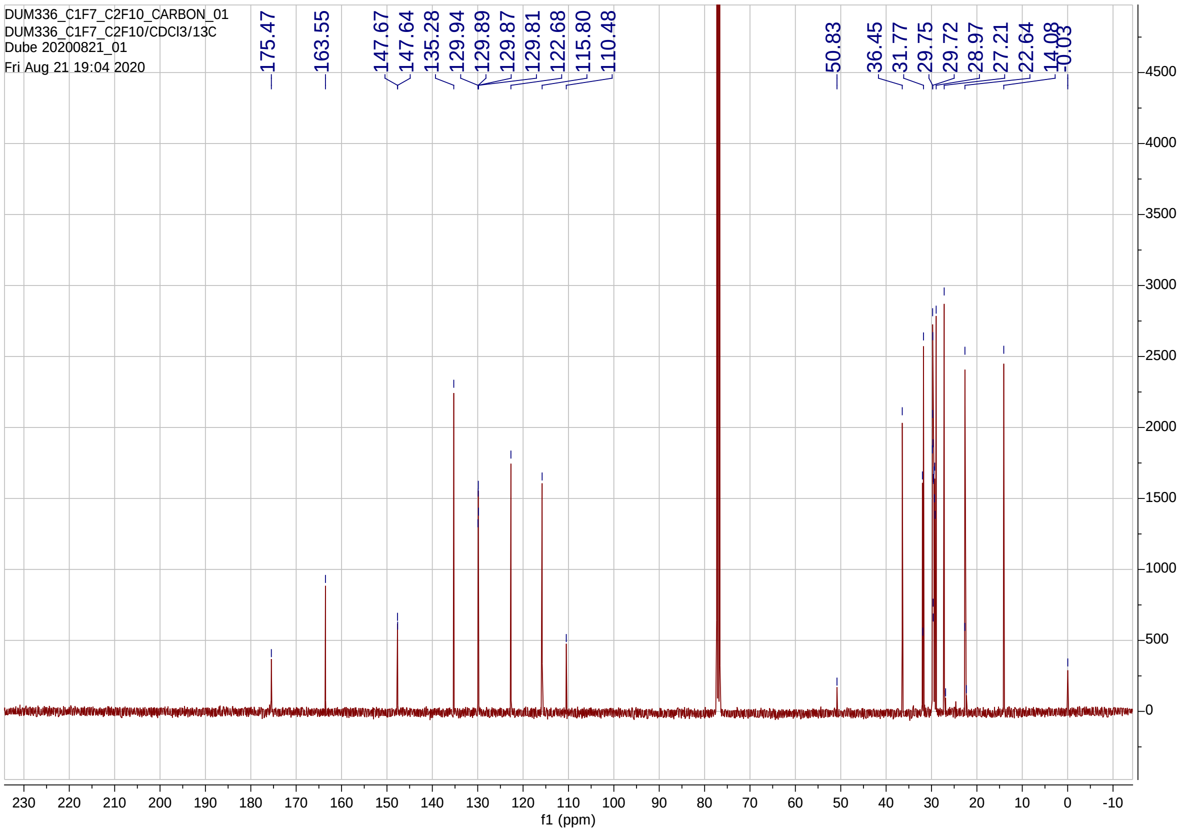

Figure S13: ^{13}C NMR spectrum of 6-[10(Z)-heptadecenyl] anacardic acid (**2**) in $CDCl_3$ (400 MHz).

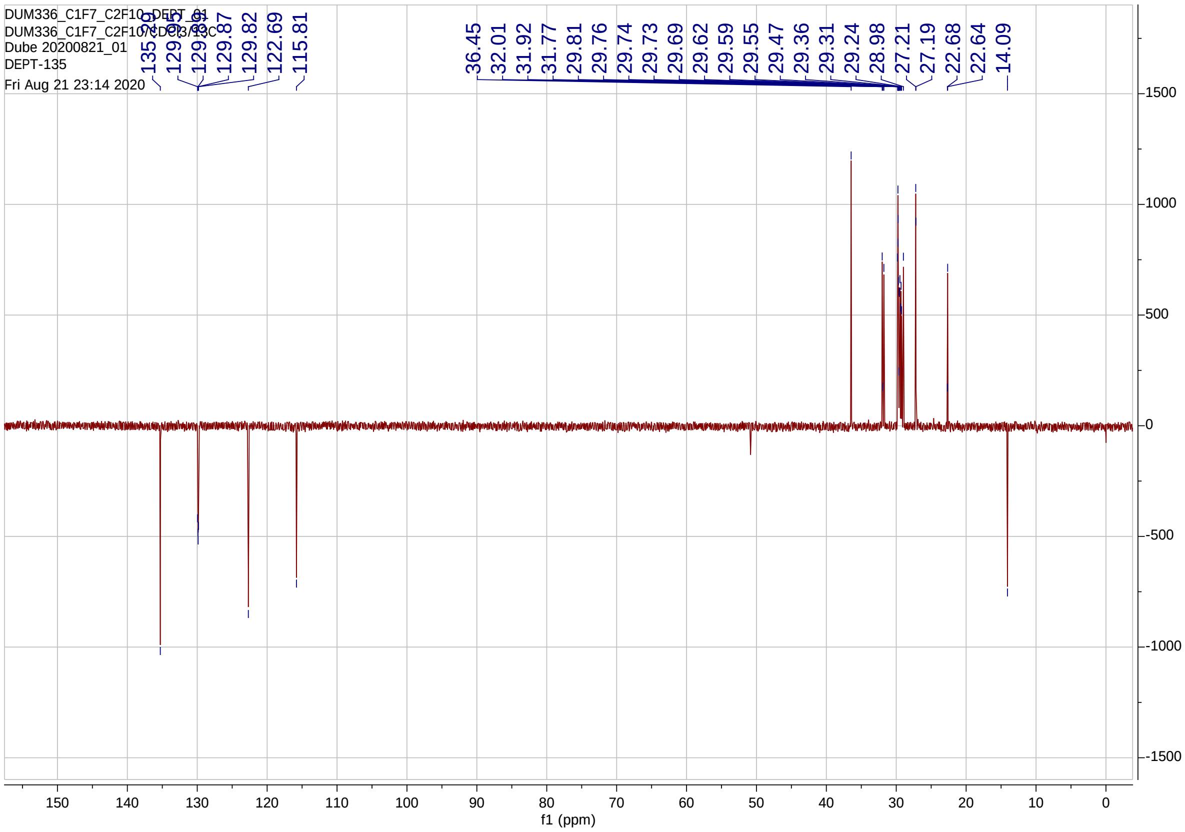

Figure S14: DEPT 135 spectrum of 6-[10(Z)-heptadecenyl] anacardic acid (**2**) in $CDCl_3$ (400 MHz).

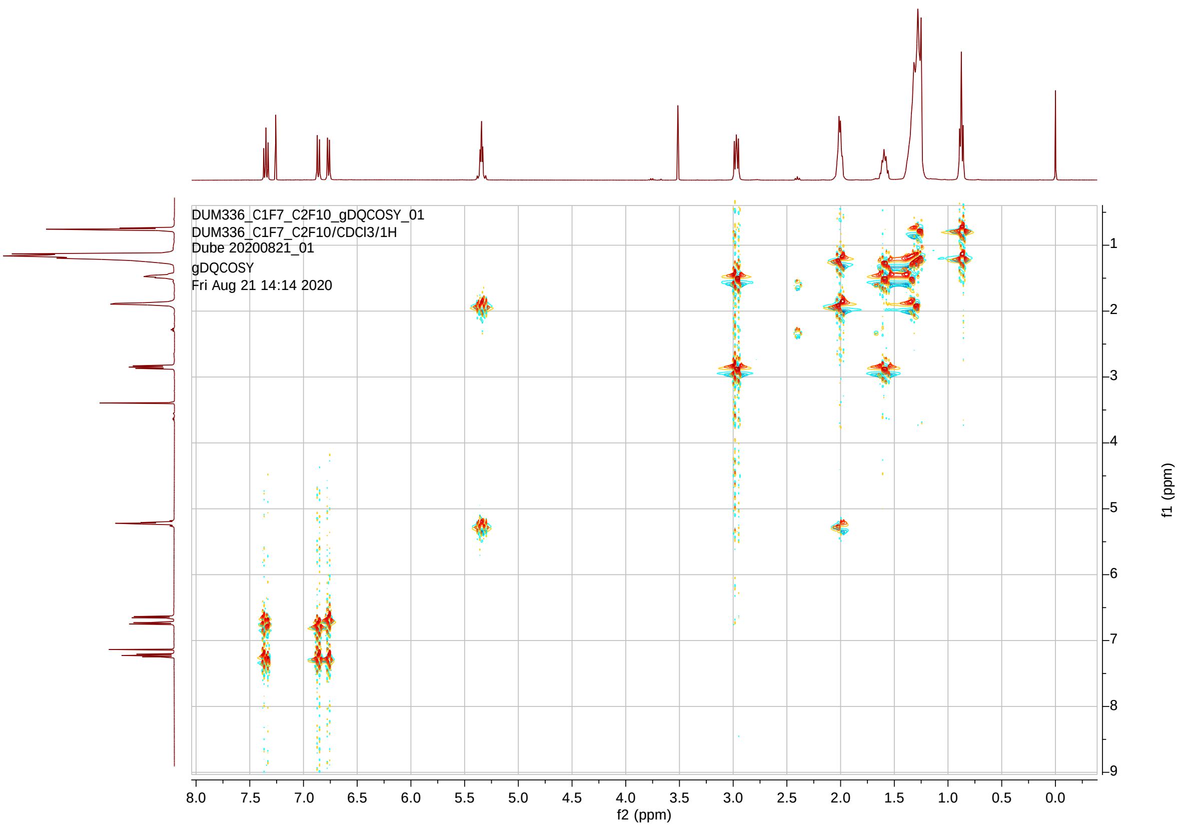

Figure S15: gDQCOSY spectrum of 6-[10(Z)-heptadecenyl] anacardic acid (**2**) in $CDCl_3$ (400 MHz).

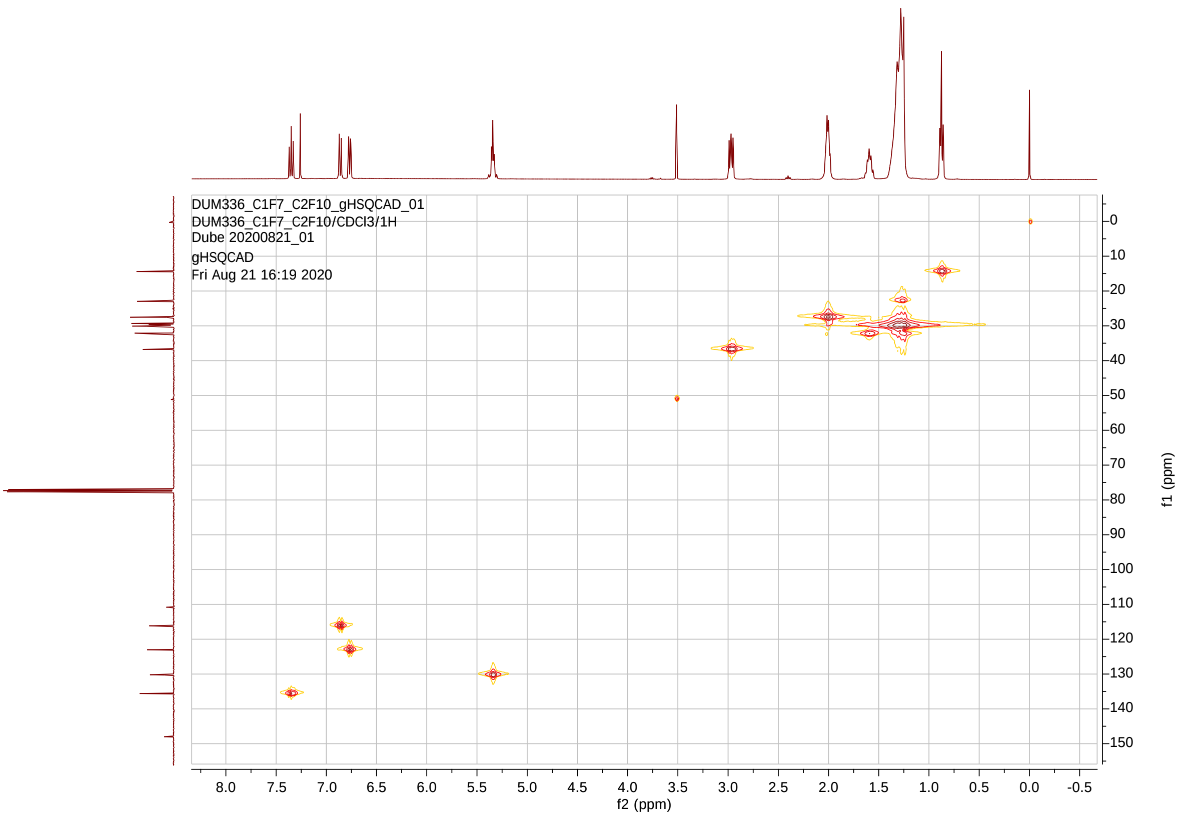

Figure S16: gHSQC spectrum of 6-[10(Z)-heptadecenyl] anacardic acid (**2**) in $CDCl_3$ (400 MHz).

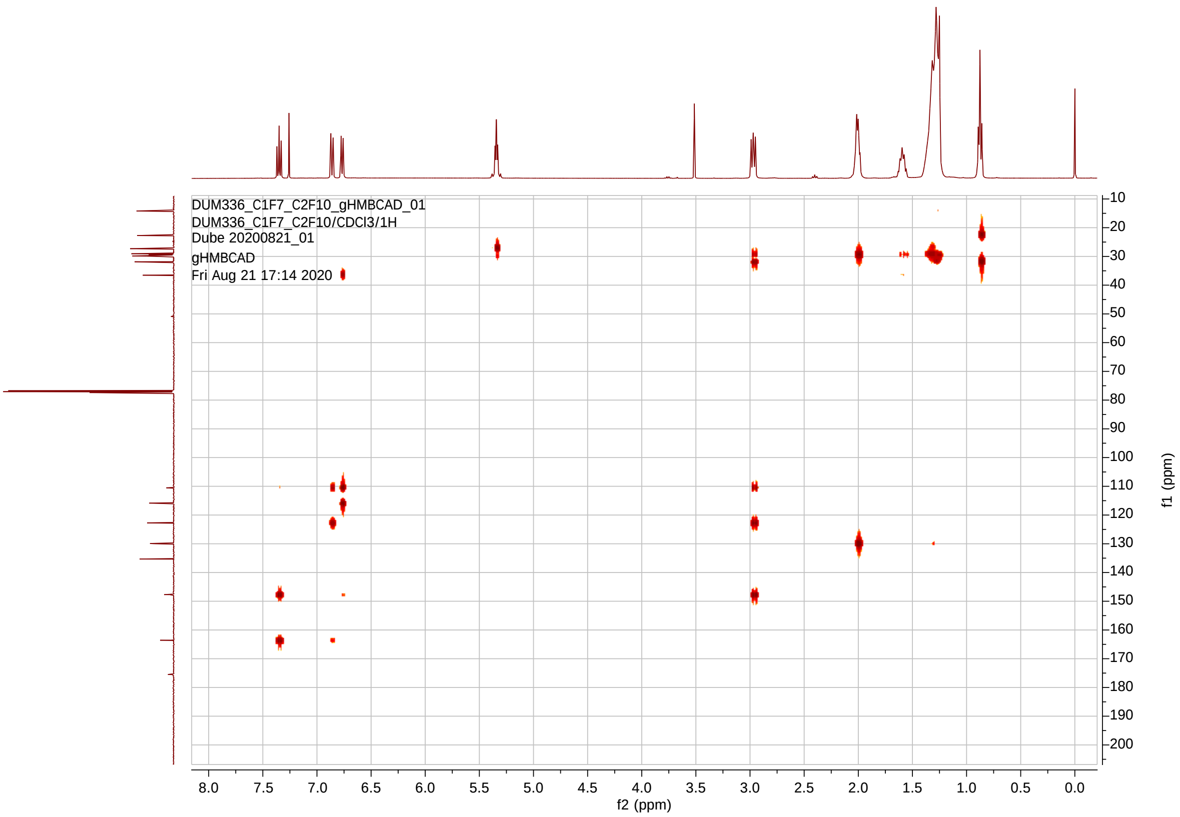

Figure S17: gHMBC spectrum of 6-[10(Z)-heptadecenyl] anacardic acid (**2**) in $CDCl_3$ (400 MHz).

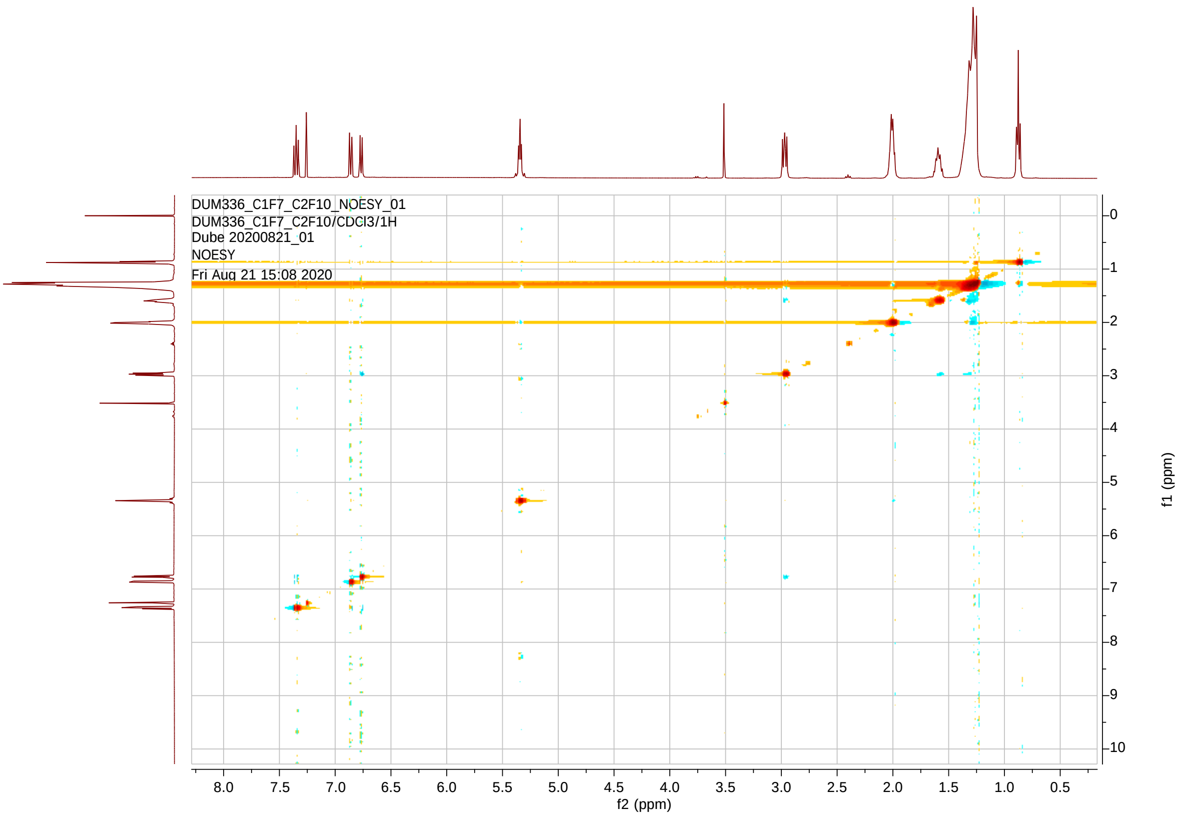

Figure S18: NOESY spectrum of 6-[10(Z)-heptadecenyl] anacardic acid (**2**) in $CDCl_3$ (400 MHz).

Figures S19 - S27: HR-MS, 1D and 2D NMR of 3-[7(*Z*)-pentadecenyl] phenol (3)

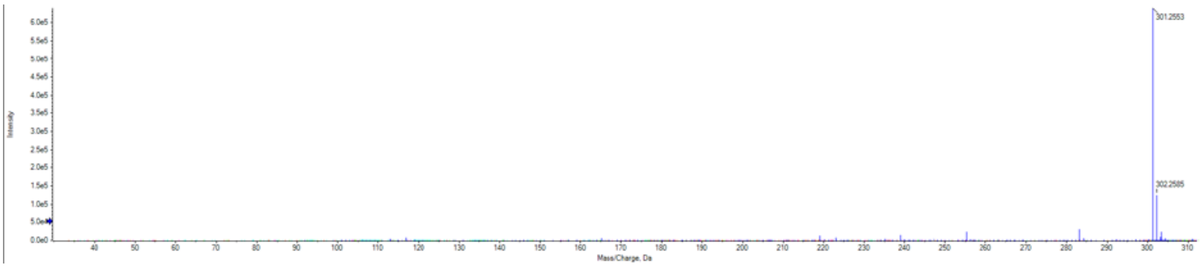

Figure S19: HR-ESI-MS spectrum (neg. mode) of 3-[7(*Z*)-pentadecenyl] phenol (**3**).

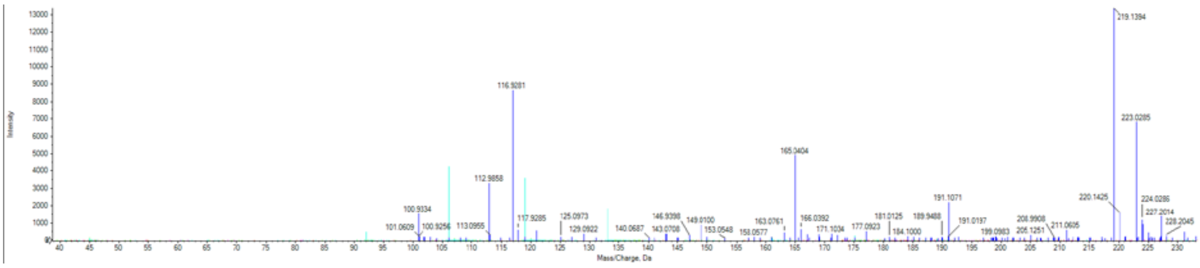

Figure S20: TOF MS^2 spectrum (neg. mode) of 3-[7(*Z*)-pentadecenyl] phenol (**3**).

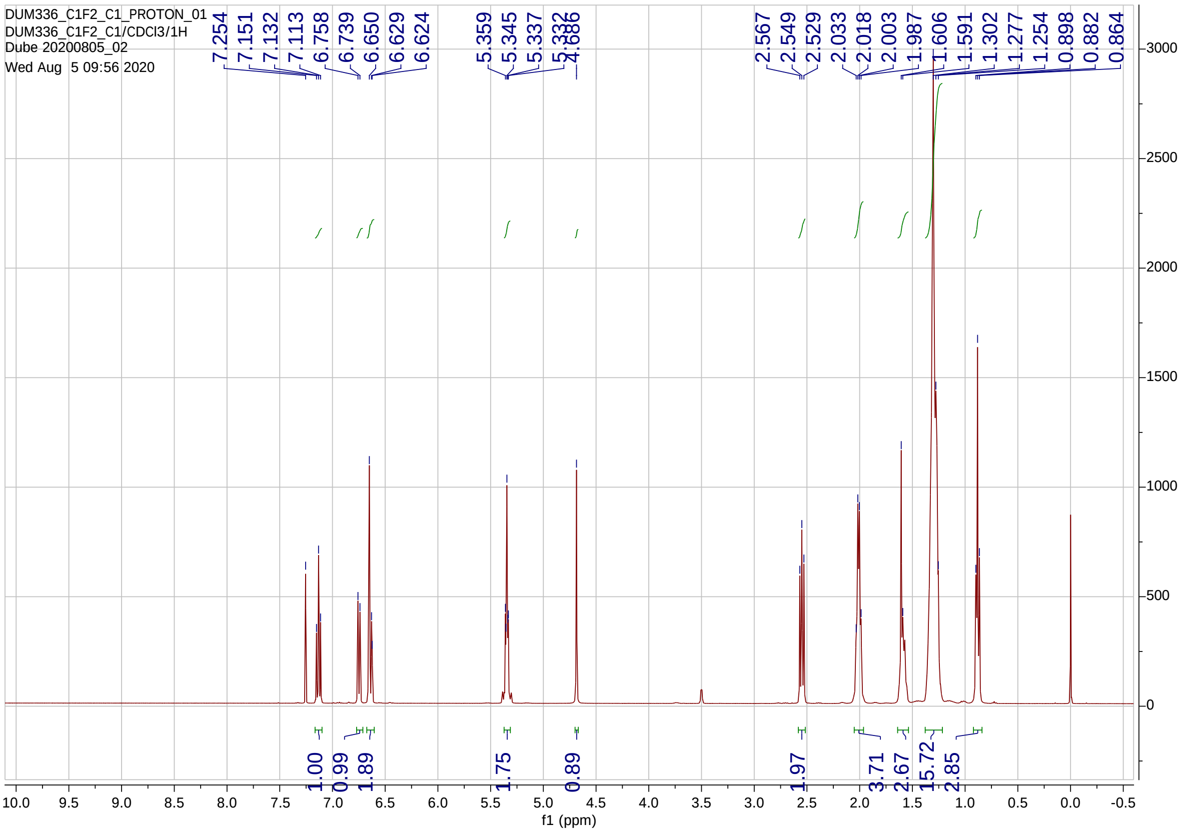

Figure S21: ^1H NMR spectrum of 3-[7(*Z*)-pentadecenyl] phenol (**3**) in $CDCl_3$ (400 MHz).

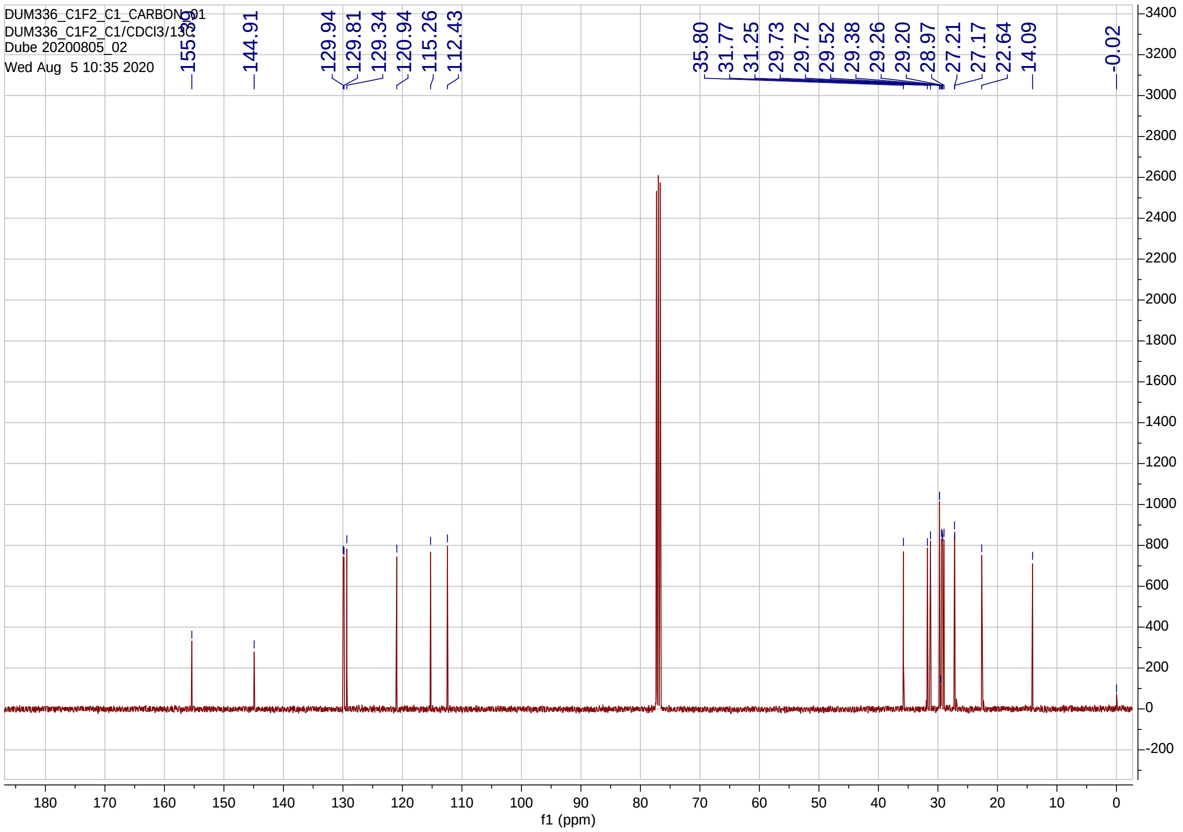

Figure S22: ^{13}C NMR spectrum of 3-[7(*Z*)-pentadecenyl] phenol (**3**) in $CDCl_3$ (400 MHz).

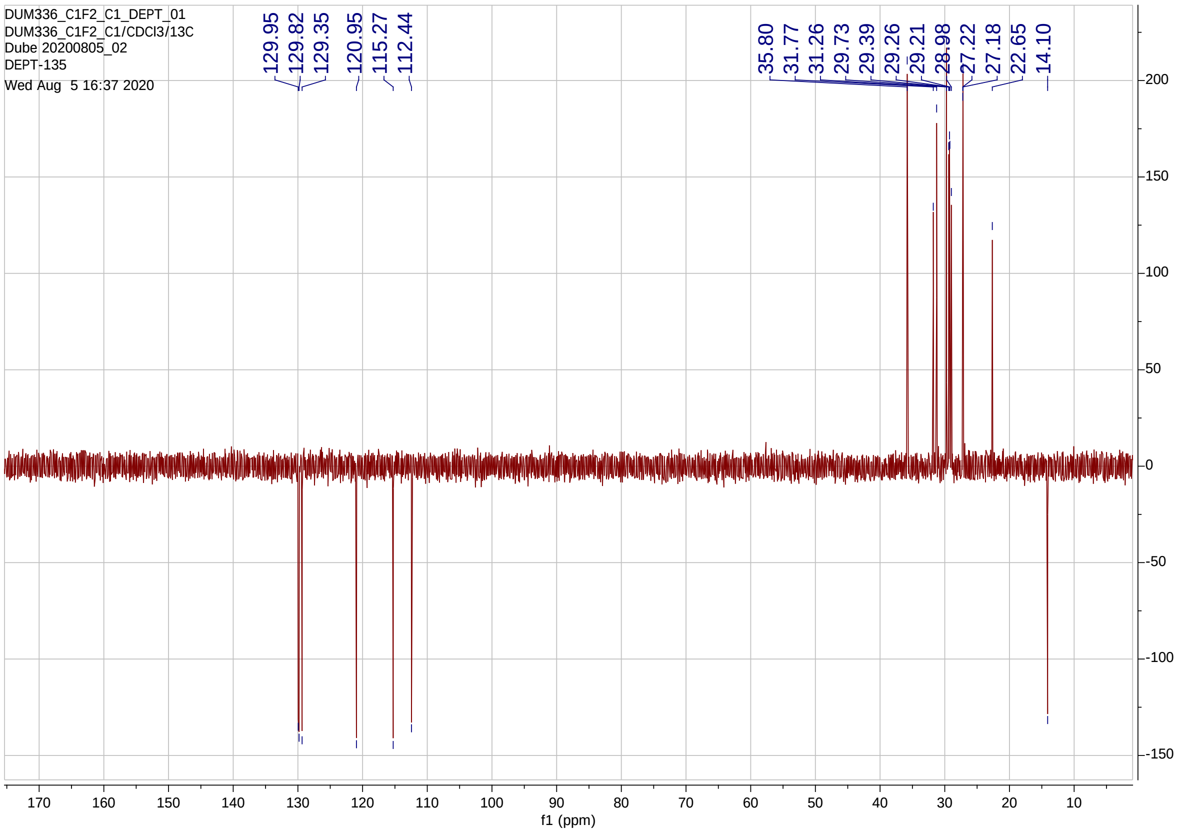

Figure S23: DEPT 135 spectrum of 3-[7(*Z*)-pentadecenyl] phenol (**3**) in $CDCl_3$ (400 MHz).

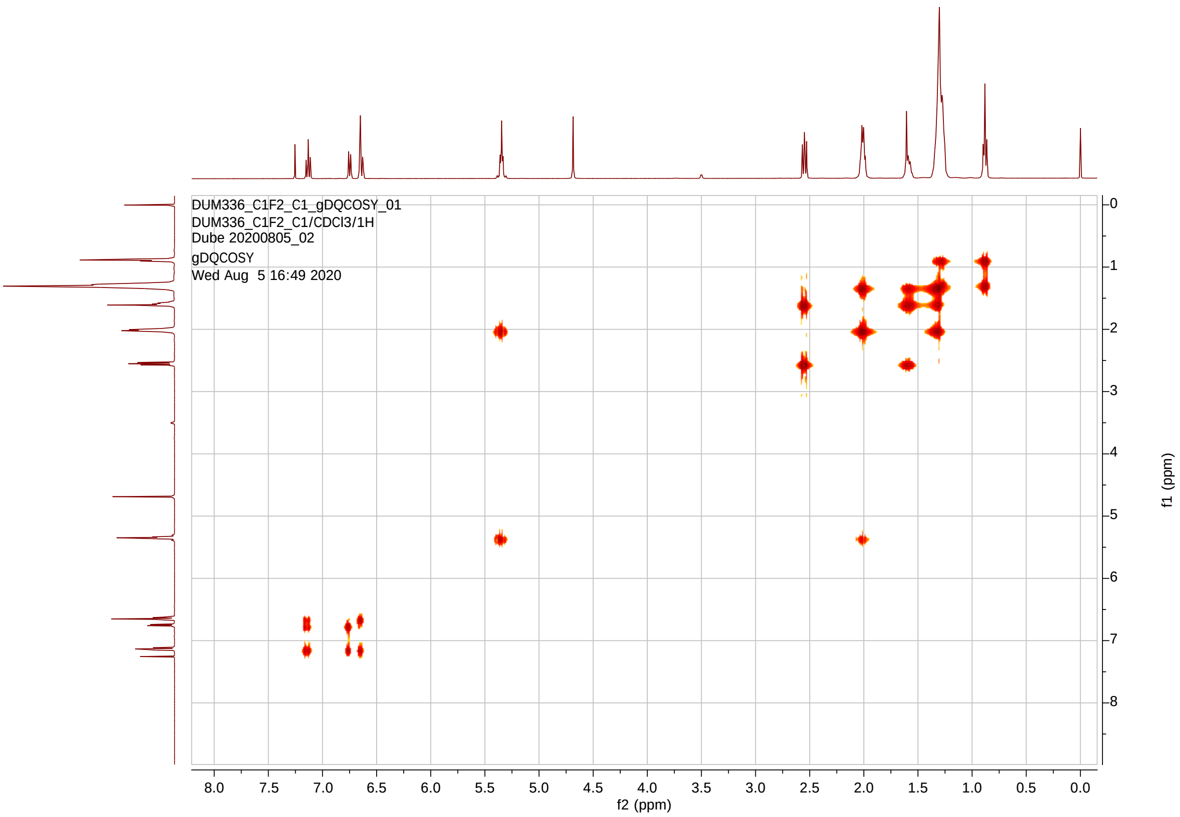

Figure S24: gDQCOSY spectrum of 3-[7(*Z*)-pentadecenyl] phenol (**3**) in $CDCl_3$ (400 MHz).

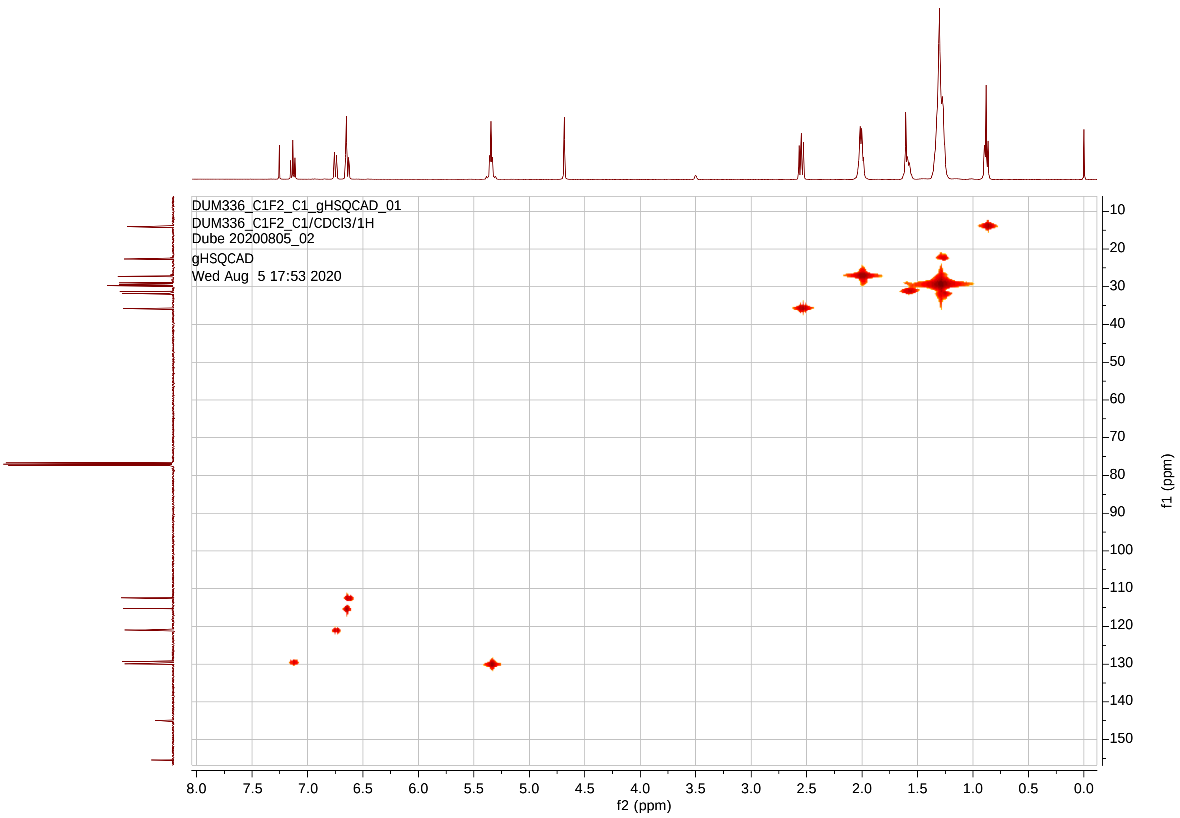

Figure S25: gHSQC spectrum of 3-[7(*Z*)-pentadecenyl] phenol (**3**) in $CDCl_3$ (400 MHz).

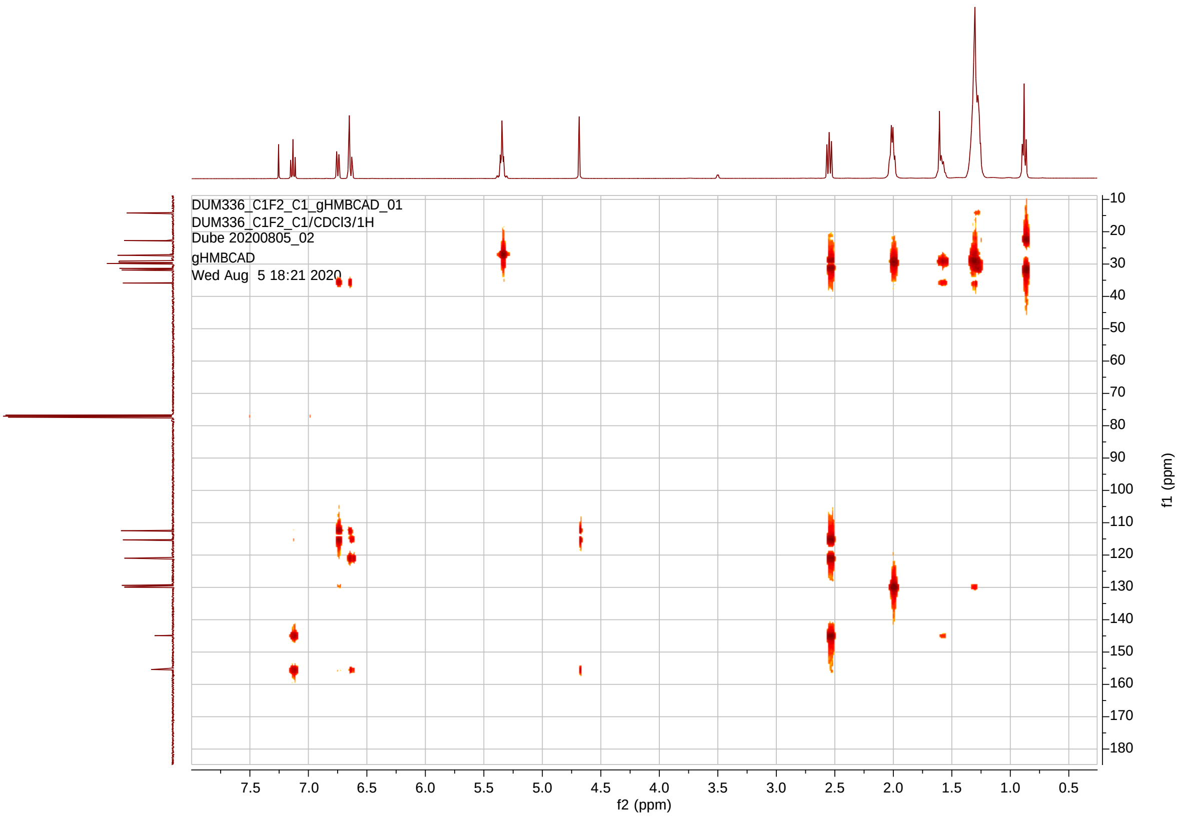

Figure S26: gHMBC spectrum of 3-[7(*Z*)-pentadecenyl] phenol (**3**) in $CDCl_3$ (400 MHz).

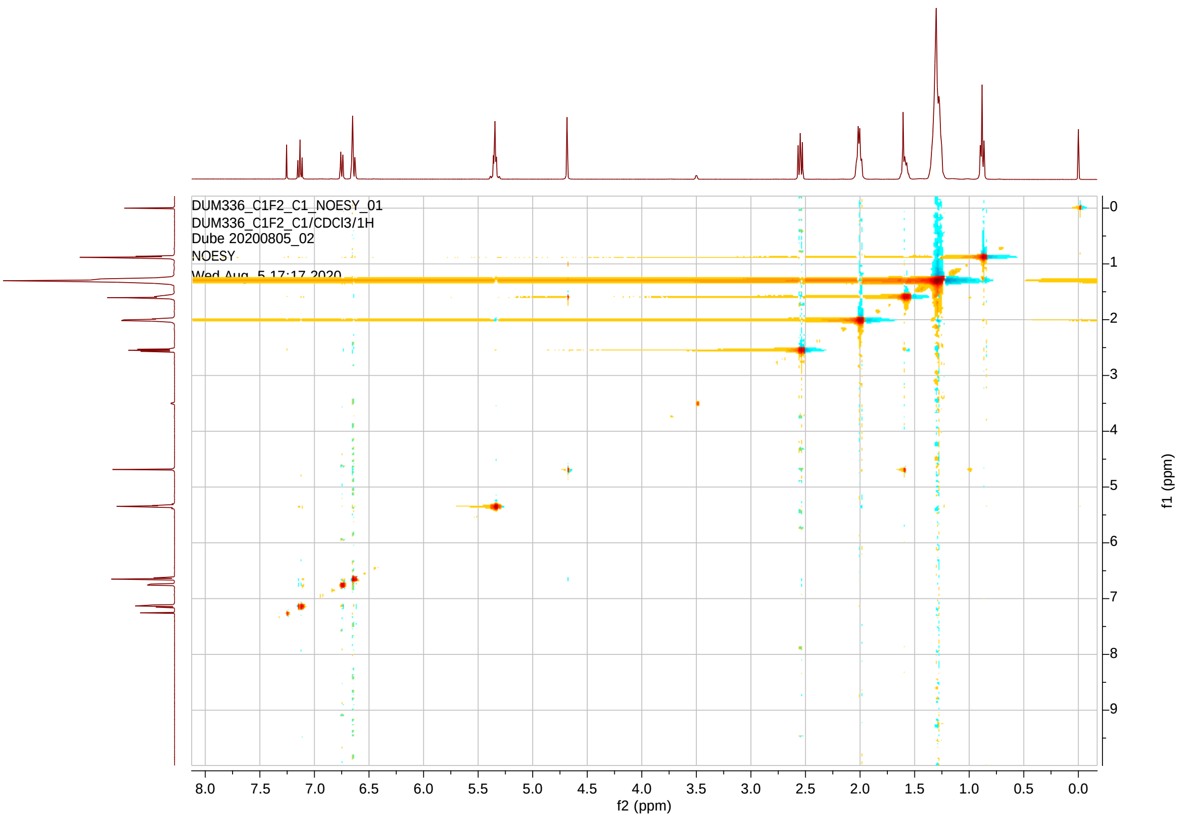

Figure S27: NOESY spectrum of 3-[7(*Z*)-pentadecenyl] phenol (**3**) in $CDCl_3$ (400 MHz).

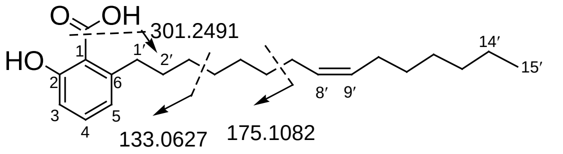

1

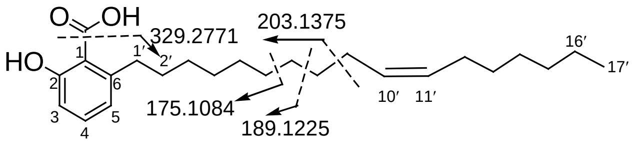

2

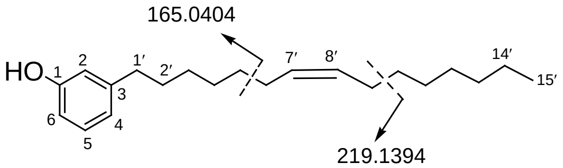

3

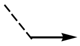 : MS^2 fragmentation

Figure S28: Important fragment ions on the TOF MS^2 spectra of compounds **1**-**3.**

Table S1: Anthelmintic activity using *C. elegans*: *Ozoroa insignis* plant parts (80 % methanol extract; test concentration 500 μg/mL; positive control ivermectin 10μg/mL).

**Phase**	**Activity % ***
Fruit extract	91.73 ± 6.05
Root extract	35.11 ± 2.91
Leaves extract	17.58 ± 3.28
Stem extract	16.42 ± 7.86
Ivermectin	98.77 ± 1.74
2% DMSO	1.91 ± 1.38

*Activity % based on three replicates.

Table S2: Anthelmintic activity using *C. elegans*: fractions from partition of *Ozoroa insignis* fruit extract (80 % methanol); test concentration 500 μg/mL; positive control ivermectin 10μg/mL).

**Phase**	**Activity %***
n-Hexane	92.54 ± 0.81
Ethyl acetate	4.54 ± 4.01
n-Butanol	4.28 ± 1.07
Aqu. residue	4.90 ± 1.70
Ivermectin	100 ± 0
2% DMSO	3.45 ± 1.48

*mortality % based on three replicates.

Table S3: ^{1}H NMR data (400 MHz, $CDCl_3$, δ in ppm) of 6-[8(*Z*)-pentadecenyl] anacardic acid (**1**), 6-[10(*Z*)-heptadecenyl] anacardic acid (**2**) and 3-[7(*Z*)-pentadecenyl] phenol (**3**) including HMBC.

**Position**	**1**		**Position**	**2**		**Position**	**3**	
	**δ_H (m, *J* in Hz)**	**Key HMBC**		**δ_H (m, *J* in Hz)**	**Key HMBC**		**δ_H (m, *J* in Hz)**	**Key HMBC**
**2**	–	_	**2**	–	_	**2**	6.65 (1H, brs)	1, 1′, 3
**3**	6.87 (1H, d, 8.4)	1, 5	**3**	6.86 (1H, d, 8.3)	1, 5	**3**	–	_
**4**	7.36 (1H, t, 8.0)	2, 6	**4**	7.35 (1H, t, 8.0)	2, 6	**4**	6.75 (1H, d, 7.6)	2, 6
**5**	6.77 (1H, d, 7.6)	1, 1′, 3	**5**	6.77 (1H, d, 7.4)	1, 1′, 3	**5**	7.13 (1H, t, 7.7)	1, 3
**6**	–	_	**6**	–	_	**6**	6.64 (1H, d, 8.3)	2, 4
**1′**	2.97 (2H, t, 7.6)	1, 5, 6	**1′**	2.97 (2H, t, 7.6)	1, 5, 6	**1′**	2.55 (2H, t, 7.2)	2, 3, 4, 2′, 3′
**2′**	1.61 (1H, m)	–	**2′**	1.60 (1H, m)	–	**2′**	1.61 (1H, m)	1′, 2′, 3′
**3′–6′**	1.29 (8H, brs)	_	**3′–8′**	1.29 (8H, brs)	_	**3′–5′**	1.30 (6H, brs)	_
**7′**	2.04 (2H, m)	8′	**9′**	2.00 (2H, m)	8′	**6′**	2.00 (2H, m)	8′, 4′
**8′**	5.35 (1H, m)	10′	**10′**	5.34 (1H, m)	10′	**7′**	5.34 (1H, m)	5′, 6′
**9′**	5.35 (1H, m)	7′	**11′**	5.34 (1H, m)	7′	**8′**	5.34 (1H, m)	9′, 10′
**10′**	2.04 (2H, m)	9′	**12′**	2.00 (2H, m)	9′	**9′**	2.00 (2H, m)	7′, 11′
**11′–13′**	1.29 (8H, brs)	_	**13′–14′**	1.27 (6H, brs)	_	**10′–12′**	1.30 (6H, brs)	_
**14′**	1.29 (2H, brs)	_	**15′**	1.27 (2H, brs)	_	**13′**	1.28 (2H, brs)	_
**15′**	0.88 (3H, t, 7.0)	13′	**16′**	1.27 (2H, brs)	13′	**14′**	1.27 (2H, m)	_
**-COO<u>H</u>**	11.04 (1H, s)	_	**17′**	0.88 (3H, t, 6.7)	_	**15′**	0.88 (3H, t, 6.8)	13′, 14′
		_	**-COO<u>H</u>**		_			

Table S4: ^{13}C NMR data (100 MHz, $CDCl_3$, δ in ppm) of 6-[8(*Z*)-pentadecenyl] anacardic acid (**1**), 6-[10(*Z*)-heptadecenyl] anacardic acid (**2**) and 3-[7(*Z*)-pentadecenyl] phenol (**3**).

**Position**	**1**	**Position**	**2**	**Position**	**3**
	**δ_C (m)**		**δ_C (m)**		**δ_C (m)**
**1**	107.5 (s)	**1**	110.5 (s)	**1**	155.4 (s)
**2**	163.6 (s)	**2**	163.6 (s)	**2**	115.3 (d)
**3**	115.8 (d)	**3**	115.8 (d)	**3**	144.9 (d)
**4**	135.3 (d)	**4**	135.3 (d)	**4**	120.9 (d)
**5**	122.7 (d)	**5**	122.7 (d)	**5**	129.3 (d)
**6**	147.6 (s)	**6**	147.6 (s)	**6**	112.4 (d)
**1′**	36.5 (t)	**1′**	36.5 (t)	**1′**	35.8 (t)
**2′**	32.0 (t)	**2′**	32.0 (t)	**2′**	31.2 (t)
**3′–6′**	29.0 – 29.8 (t)	**3′–8′**	29.0 – 29.8 (t)	**3′–5′**	29.0 – 29.8 (t)
**7′**	27.2 (t)	**9′**	27.2 (t)	**6′**	27.2 (t)
**8′**	129.9 (d)	**10′**	129.9 (d)	**7′**	129.9 (d)
**9′**	129.9 (d)	**11′**	129.8 (d)	**8′**	129.8 (d)
**10′**	27.2 (t)	**12′**	27.2 (t)	**9′**	27.2 (t)
**11′–12′**	29.0 – 29.8 (t)	**13′–14′**	29.0 – 29.8 (t)	**10′–12′**	29.0 – 29.8 (t)
**13′**	31.8 (t)	**15′**	31.8 (t)	**13′**	31.8 (t)
**14′**	22.6 (t)	**16′**	22.6 (t)	**14′**	22.5 (t)
**15′**	14.1 (q)	**17′**	14.1 (q)	**15′**	14.1 (q)
**-COOH**	175.4 (s)	**-COOH**	175.5 (s)		

Table S5: Anthelmintic activity using *C. elegans*: anthelmintic activity of 6-[8(*Z*)-pentadecenyl] anacardic acid (**1**), 6-[10(*Z*)-heptadecenyl] anacardic acid (**2**) and 3-[7(*Z*)-pentadecenyl] phenol (**3**).

***conc.* (µg/mL)**	**Activity %* (1)**	**µM (1)**	**Activity %* (2)**	**µM (2)**	**Activity %* (3)**
500	100 ± 0	1440.00	100 ± 0	1334.80	0.79 ± 1.12
250	100 ± 0	721.40	97.73 ± 1.72	667.40	n. t.
125	97.01 ± 0.22	360.70	94.39 ± 5.36	333.70	n. t.
62.5	78.19 ± 9.99	180.30	73.98 ± 3.12	166.80	n. t.
31.25	63.42 ± 7.29	90.18	47.41 ± 3.37	83.43	n. t.
15.625	46.40 ± 10.02	45.09	24.78 ± 2.28	41.71	n. t.
Ivermectin (10µg/mL)	99.07 ± 1.31		95.59 ± 0.71		98.45 ± 1.26
2% DMSO	0.95 ± 1.35		0		1.36 ± 0.97

*Activity % based on three replicates.

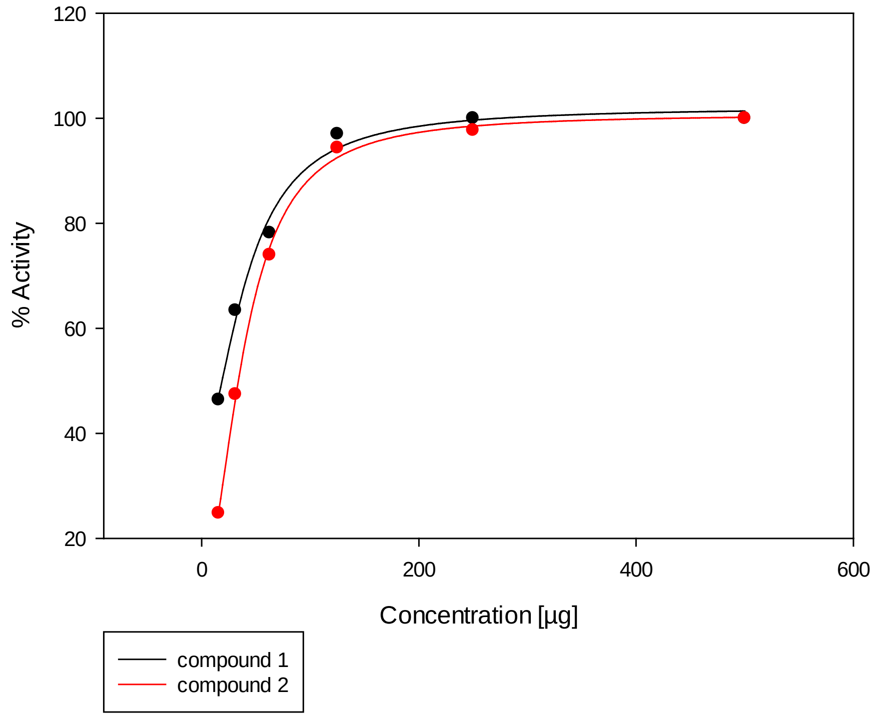

Figure S29: Graph for LC_{50} of 6-[8(Z)-pentadecenyl] anacardic acid (**1**) and 6-[10(Z)-heptadecenyl] anacardic acid (**2**) (testorganism: *C. elegans*).

Table S6: LC_{50} values of 6-[8(Z)-pentadecenyl] anacardic acid (**1**), 6-[10(Z)-heptadecenyl] anacardic acid (**2**) (testorganism: *C. elegans*).

**Compound**	**LC50 (µM)**
**1**	51,9 ± 10,33
**2**	93,4 ± 3,53
